# Identification of the Cysteine Protease Legumain as a Potential Chronic Hypoxia-Specific Multiple Myeloma Target Gene

**DOI:** 10.3390/cells11020292

**Published:** 2022-01-15

**Authors:** Ada-Sophia Clees, Verena Stolp, Björn Häupl, Dominik C. Fuhrmann, Frank Wempe, Marcel Seibert, Sarah Weber, Antje Banning, Ritva Tikkanen, Richard Williams, Bernhard Brüne, Hubert Serve, Frank Schnütgen, Ivana von Metzler, Nina Kurrle

**Affiliations:** 1 Department of Medicine, Hematology/Oncology, University Hospital Frankfurt, Goethe-University Frankfurt, 60590 Frankfurt, Germany; aclees@posteo.de (A.-S.C.); stolp@med.uni-frankfurt.de (V.S.); b.haeupl@dkfz-heidelberg.de (B.H.); wempe@em.uni-frankfurt.de (F.W.); seibert@med.uni-frankfurt.de (M.S.); Sarah.Weber@kgu.de (S.W.); serve@em.uni-frankfurt.de (H.S.); schnuetgen@em.uni-frankfurt.de (F.S.); 2 German Cancer Consortium (DKTK), Partner Site Frankfurt/Mainz, and German Cancer Research Center (DKFZ), 69120 Heidelberg, Germany; bruene@pathobiochemie1.de; 3 Frankfurt Cancer Institute, Goethe-University Frankfurt, 60596 Frankfurt, Germany; 4 Institute of Biochemistry I, Faculty of Medicine, Goethe-University Frankfurt, 60590 Frankfurt, Germany; fuhrmann@med.uni-frankfurt.de; 5 Institute of Biochemistry, Faculty of Medicine, University of Giessen, 35392 Giessen, Germany; Antje.Banning@biochemie.med.uni-giessen.de (A.B.); ritva.tikkanen@biochemie.med.uni-giessen.de (R.T.); 6 Patrick G Johnston Centre for Cancer Research, Queen’s University Belfast, 97 Lisburn Road, Belfast BT9 7AE, UK; rich.williams@qub.ac.uk

**Keywords:** multiple myeloma, chronic hypoxia, asparaginyl endopepdidase (AEP), legumain, glycolysis

## Abstract

Multiple myeloma (MM) is the second most common hematologic malignancy, which is characterized by clonal proliferation of neoplastic plasma cells in the bone marrow. This microenvironment is characterized by low oxygen levels (1–6% O_2_), known as hypoxia. For MM cells, hypoxia is a physiologic feature that has been described to promote an aggressive phenotype and to confer drug resistance. However, studies on hypoxia are scarce and show little conformity. Here, we analyzed the mRNA expression of previously determined hypoxia markers to define the temporal adaptation of MM cells to chronic hypoxia. Subsequent analyses of the global proteome in MM cells and the stromal cell line HS-5 revealed hypoxia-dependent regulation of proteins, which directly or indirectly upregulate glycolysis. In addition, chronic hypoxia led to MM-specific regulation of nine distinct proteins. One of these proteins is the cysteine protease legumain (LGMN), the depletion of which led to a significant growth disadvantage of MM cell lines that is enhanced under hypoxia. Thus, herein, we report a methodologic strategy to examine MM cells under physiologic hypoxic conditions in vitro and to decipher and study previously masked hypoxia-specific therapeutic targets such as the cysteine protease LGMN.

## 1. Introduction

Multiple Myeloma (MM) is the second most frequent hematologic malignancy with an annual incidence of 3.6–6.3/100,000, an estimated annual total number of approximately 160,000 new cases and 106,000 deaths worldwide [1,2]. MM is marked by clonal proliferation of neoplastic plasma cells within the bone marrow (BM) microenvironment (BMME). These cells initiate a number of disease processes that affect among others hematopoiesis, the bones and the kidneys. MM cells secrete monoclonal immunoglobulins, and by infiltration, paracrine and endocrine mechanisms suppress normal hematopoiesis and hyperactivate bone-resorbing osteoclasts. As a result, MM often presents with a disseminated osteolytic and osteoporotic bone disease, hypercalcemia, reduced blood cell counts and immunodeficiency, and—less frequently—with a destructive kidney disease that results in renal insufficiency. Although great progress has been made with respect to therapy, MM remains an incurable disease [3,4].

The pathophysiology of MM is strongly influenced by the BMME [5,6,7]. Mutual interactions between plasma cells and cells of the microenvironment, such as osteoclasts and endothelial cells, induce BMME adaptation that support growth and survival of MM cells and frequently confer drug resistance [8,9,10]. In addition, within the BMME, cells are adapted to oxygen levels varying from 1–6% oxygen (O_2_) [11,12], which is an environmental cue that is commonly termed as hypoxia. Particularly in solid tumors, hypoxia is an established factor promoting the invasive and metastatic behavior of cancer cells [13,14,15,16]. Likewise, hypoxia in the BM is a physiological feature of both healthy and malignant cells and was shown to mediate therapeutic resistance in hematologic malignancies, e.g., acute myeloid leukemia [13,14,15,16]. Specifically for MM, hypoxia has been reported to promote a stem cell-like phenotype, to confer resistance to various inhibitors, such as the proteasome inhibitor bortezomib, and to stimulate migration and homing of malignant plasma cells in vitro and in vivo [17,18,19,20,21,22].

Hypoxia-inducible factors (HIFs) have been found to be major regulators of the cellular adaptation to low oxygen levels. HIF are heterodimeric transcription factors consisting of an oxygen-regulated α-subunit and a constitutively expressed β subunit [23], which are known to regulate the expression of more than 100 so-far-identified target genes such as the glucose transporter, solute carrier family member 1 (*SLC2A1*/*GLUT1*) [24,25]. High expression levels of HIFs have been correlated with tumor growth and resistance and are thereby considered to be markers of poor prognosis in solid cancers [26]. In MM, various studies have shown that neoplastic plasma cells from newly diagnosed MM patients exhibit an increase in HIF1α and HIF2α regulated pathways compared to cells isolated from healthy donors [21,22,27] and that inhibition of HIF1α might provide a strategy to prevent hypoxia-induced adaptations in malignant plasma cells [28,29,30,31,32].

Despite these interesting results obtained in MM cells under low oxygen levels in vitro, the interpretation of these data remains difficult. Investigations of MM cells cultured under physiologic hypoxic conditions are scarce, and they are usually performed under conditions that do not allow researchers to discriminate short-term versus long-term effects of changes in oxygen tension [17,18,19,20,21,22,33,34,35]. To address this issue, herein, we report a strategy to examine MM cells under physiological hypoxic conditions. This allowed us to identify novel proteins that are regulated and seem to play a role in MM cell proliferation and survival predominantly in hypoxia. The cysteine protease legumain (LGMN) is one of these proteins.

## 2. Materials and Methods

### 2.1. Cell Culture

Human MM cell lines (RPMI-8226, LP-1, U266, OPM-2 and KMS-12-BM) were obtained from the DSMZ (Deutsche Sammlung von Mikroorganismen und Zellkulturen GmbH, Braunschweig, Germany). The human bone marrow stromal cell line HS-5 was obtained from ATCC (American Type Culture Collection, Manassas, VA, USA). Cells were cultured in RPMI 1640 medium (Gibco/Thermo Fisher Scientific, Darmstadt, Germany #21875034) supplemented with 10% fetal calf serum (FCS, Sigma-Aldrich, Taufkirchen, Germany) and 100 IU/mL penicillin and 100 mg/mL streptomycin (P/S, Gibco/Thermo Fisher Scientific, Karlsruhe, Germany #15140122) at 37 °C in a humidified 5% CO_2_ incubator. The semi-adherent cell lines RPMI-8226, LP-1 and U266 had to be detached carefully from the cell culture plate using a cell scraper before splitting. The suspension cell lines OPM-2 and KMS-12-BM were mixed directly by pipetting up and down. Lenti-X 293T cells (Takara Bio Europe SAS, Saint-Germain-en-Laye, France, #Z2180N) were cultured in Dulbecco’s modified Eagel’s medium (DMEM 4.5 g/L D-glucose, Gibco/Thermo Fisher Scientific, #41965-039) and supplemented with 10% FCS and 100 IU/mL penicillin and 100 mg/mL streptomycin (P/S, Gibco/Thermo Fisher Scientific, #15140122) at 37 °C in a humidified 5% CO_2_ incubator. To simulate physiologic hypoxic conditions, cells were cultured in a hypoxia chamber (Biospherix, Parish, NY, USA, X3 Xvivo System). Cells were kept at 37 °C under 1% O_2_, 5% CO_2_ and 94% N_2_. This condition will further be termed hypoxia. Liquids used in the chamber, e.g., 1× phosphate buffered saline (1× PBS) and RPMI 1640 medium were adapted to hypoxic condition over night for 12 h. Human MM cell lines stably expressing spCas9 were generated by lentiviral transduction (see Section 2.9) by using the plasmid LentiCas9-Blast (Addgene, Watertown, MA, USA #52962, [36]).

### 2.2. Antibodies

Goat polyclonal antibody against human legumain (LGMN) was used for Western blotting, and immunofluorescence was purchased from R&D Systems Inc. (Bio-Techne GmbH, Wiesbaden, Germany, #AF2199). Rabbit monoclonal antibodies against hexokinase 2 (HK2, #2867), lactate dehydrogenase (LDHA, #3582), β-tubulin (#2128) and β-Actin (#4970) were purchased from Cell Signaling Technology Europe B.V. (Frankfurt, Germany). A mouse monoclonal antibody against Vinculin (#ab18058) was purchased from Abcam (Cambridge, UK). For HIF detection, a polyclonal rabbit antibody against HIF-1α (#NB100-134) from Novus biologicals (Bio-Techne GmbH, Wiesbaden, Germany) and a polyclonal goat antibody for HIF-2α (#AF2886) from R&D systems Inc. were used. A rabbit antibody against nucleolin was obtained from Santa Cruz Biotechnology (Heidelberg, Germany, #sc-13057). The primary antibody used for immunofluorescence was detected with a Cy3-conjugated rabbit anti-goat antibody (Jackson ImmunoResearch, Ely, UK). The primary antibodies used for Western blotting were detected with an HRP-conjugated goat anti-rabbit (Dako, Agilent Technologies, Waldbronn, Germany), donkey anti-goat antibody (Santa Cruz Biotechnology, Heidelberg, Germany) or anti-mouse (LI-COR Biosciences, Bad Homburg, Germany) antibody.

### 2.3. RNA Extraction, cDNA Preparation and Real-Time PCR Measurement

RPMI-8226, LP-1, U266, OPM-2, KMS-12-BM and HS-5 cells were cultured in 21% O_2_ and 1% O_2_ and harvested after 0, 1, 3, 5 and 7 days or 0, 1 and 7 days. RNA was isolated using the NucleoSpin RNA purification kit (Macherey-Nagel, Dueren, Germany, #740962.20) according to the manufacturer’s instructions. An amount of 3 μg of RNA was reverse-transcribed with 2 μM oligo(dT) primers (NEB, # S1327S), 2 μM random primers (New England Biolabs, Frankfurt, Germany,, # S1330S), dNTPs (New England Biolabs, #N0446S), RNAse inhibitor (Thermo Fisher Scientific, #10777019) and 200 units Moloney murine leukemia virus reverse transcriptase (ProtoScript II reverse transcriptase, New England Biolabs, #M0368) in a total volume of 20 μL. Real-time PCRs (Opticon 2 reader, MJ research, now BioRad, Dreieich, Germany) were performed in triplicates with 2.5 μL of 5-fold diluted cDNA in a 25 μL reaction using SYBR^®^ Green JumpStart™ Taq ReadyMix™ (Sigma-Aldrich, #S4438). The annealing temperature was 60 °C for all PCR reactions. Primers are listed in Table 1. The reference gene TATA-box-binding protein (*TBP*) was used for normalization.

### 2.4. Stable Isotope Labeling with Amino Acids in Cell Culture (SILAC) Labeling and Cell Lysis

SILAC labeling was performed using SILAC RPMI (Thermo Fisher Scientific, #88365) supplemented with 10% dialyzed FCS (Gibco/Thermo Fisher Scientific, #26400044), 100 U/mL penicillin, 100 μg/mL streptomycin (Gibco/Thermo Fisher Scientific, #15140122) and the respective SILAC amino acids (all from Cambridge Isotopes, Tewksbury, MA, USA). “Light” SILAC medium contained [^12^C_6_^14^N_4_]-L-arginine and [^12^C_6_^14^N_2_]-L-lysine. “Heavy” SILAC medium contained [^13^C_6_^15^N_4_]-L-arginine and [^13^C_6_^15^N_2_]-L-lysine. In “heavy” SILAC medium 2 µM/L L-proline was added (Thermo Fisher Scientific, #88211). Cells cultured under normoxic conditions received “heavy” amino acids for 6 passages before cell lysis. Cells receiving “light” amino acids were cultured under 21% O_2_ for 3 passages and under 1% O_2_ for another 3 passages. Overall, “light” labeled cells spent 7 days under hypoxic conditions. The MM cell lines RPMI-8226, LP-1, U266, KMS-12-BM, OPM-2 as well as the bone marrow stromal cell line HS-5 were labeled both under normoxic and hypoxic conditions.

For cell lysis, cells were initially washed with cold 1× PBS. Hypoxia-treated cells were washed with 1%-O_2_ adapted 1× PBS. Hereafter, cells were lysed in 0.5% Nonidet P-40 buffer (50 mM Tris-HCl pH 7.5, 150 mM NaCl, 1 mM Na_3_VO_4_, 5 mM NaF) freshly supplemented with protease inhibitors (cOmplete™, Mini, EDTA-free Protease Inhibitor Cocktail, Roche/Merck, Darmstadt, Germany, #11836170001). Cells were kept on ice for 30 min and centrifuged at 21,380× *g* for 10 min at 4 °C. Protein concentration was analyzed using Bradford (ROTI-Quant, Carl Roth, Karlsruhe, Germany, #K015.1). Samples of light and heavy were mixed 1:1. All buffers were prepared with HPLC-grade water (Sigma-Aldrich, #270733) in glass ware cleaned with acetone before use. Tubes were low-binding tubes (Thermo Fisher Scientific, #90411). 4× LDS-sample buffer (Thermo Fisher Scientific, #NP0008) containing 200 mM HPLC-grade DL-Dithiothreitol (DTT, Sigma-Aldrich, #D0632) was added and samples were boiled for 5 min, 95 °C. For mass spectrometry (MS) analysis, 3 replicates were used for all cell lines except OPM-2.

### 2.5. Global Proteome Analysis

For protein expression profiling, lysates from SILAC were mixed in equal amounts and separated by SDS-PAGE using precast Bis-Tris minigels (NuPAGE Novex 4–12%, Life Technologies/ Thermo Fisher Scientific). After protein staining with Coomassie Brilliant Blue (SERVA Electrophoresis GmbH, Heidelberg, Germany), each gel lane was cut into 23 slices. The separated proteins in each gel slice were reduced with DTT (Sigma-Aldrich) and alkylated with iodoacetamide (Sigma-Aldrich). After in-gel protein digestion with trypsin (SERVA Electrophoresis GmbH) over night, the peptides were extracted from the gel matrix and analyzed by liquid chromatography/tandem mass spectrometry (LC-MS/MS).

The peptide mixtures were analyzed using a quadrupole-Orbitrap hybrid mass spectrometer (Q Exactive Plus, Thermo Fisher Scientific) coupled to an EASY n-LC 1000 HPLC system (Thermo Fisher Scientific). The samples were first desalted on a trap column (20 × 0.1 mm; packed in-house with ReproSil-Pur 120 C18-AQ, 5 μm; Dr. Maisch GmbH, Ammerbuch, Germany) at 5 μL/·min in loading buffer [2% (*v*/*v*) ACN, 0.1% FA] and then separated on an analytical column (320 × 0.075 mm; packed in-house with ReproSil-Pur 120 C18-AQ, 1.9 μm; Dr. Maisch GmbH) using an 80-min linear gradient from 5% to 42% buffer B [95% (*v*/*v*) ACN, 0.1% FA] over buffer A (0.1% FA) at a flow rate of 300 nL/min. Eluting peptides were analyzed using a data-dependent acquisition scheme selecting the top 20 most abundant precursor ions (charge states 2 to 4) for higher energy collisional dissociation (HCD) with an isolation width of 1.6 m/z and an NCE setting of 28%. Survey spectra (MS) in the range of m/z 350–1600 were acquired with a resolution of 70,000 FWHM at m/z 200 and product ion spectra (MS/MS) using a resolution setting of 17,500 FWHM at m/z 200. AGC target values and maximum injection times for MS and MS/MS were set to 1 × 10^6^ in 50 ms and 5 × 10^4^ in 50 ms, respectively. Fragmented ions were excluded from isolation for 30 s.

The mass spectrometry proteomics data have been deposited to the ProteomeXchange Consortium via the PRIDE [37] partner repository with the dataset identifier PXD030239.

### 2.6. MS Data Processing

Raw data files from LC-MS/MS measurements were analyzed using the MaxQuant software (version 1.6.0.1, MPI for Biochemistry) [38]. Spectra were searched against the UniProtKB/Swiss-Prot human database containing 88,993 protein entries (downloaded November 2016) supplemented with 245 frequently observed contaminants with the Andromeda search engine [38]. Precursor and fragment ion mass tolerances were set to 6 and 20 ppm after initial recalibration, respectively. Protein N-terminal acetylation and methionine oxidation were allowed as variable modifications. Cysteine carbamidomethylation was defined as a fixed modification. Minimal peptide length was set to seven amino acids, with a maximum of two missed cleavages. The false discovery rate (FDR) was set to 1% on both the peptide and the protein level using a forward-and-reverse decoy database approach. For SILAC quantitation, multiplicity was set to 2 for double labeling (Lys + 0/Arg + 0, Lys + 8/Arg + 10) and at least 2 ratio counts were required for peptide quantitation. Both the “match between runs” and “re-quantify” options of MaxQuant were enabled.

Subsequent evaluation of MaxQuant output data was conducted with the Perseus software (version 1.6.0.7, MPI for Biochemistry) [39]. After removal of hits from the decoy database search and potential contaminants, the SILAC ratios were log2-transformed. To assign regulated proteins for each cell line, the SILAC ratios were filtered for 2-fold up- or down-regulation in at least 2 biological replicates. For statistical evaluation of hypoxia-induced protein expression changes, a one-sample *t*-test of log2-transformed SILAC ratios was conducted by applying a Benjamini-Hochberg FDR < 5% to adjust the *p*-values.

For each cell line, regulated proteins were subjected to Ingenuity Pathway Analysis (IPA summer release 2021, Qiagen, Hilden, Germany). For this, an IPA core expression analysis on the log-transformed SILAC ratios was conducted using the default software settings. The results of the canonical pathways module were matched in an IPA comparison analysis and visualized by plotting the activation Z-scores of pathways scoring in at least two cell lines.

### 2.7. Immunofluorescence

Semiadherent and adherent cells (LP-1, RPMI-8226 and HS-5) were cultured on coverslips for 24 h and suspension cells (U266, OPM-2) were attached to microscope slides (Adhesive slides Superfrost Plus, #H867.1, Carl Roth) by centrifugation (Cytospin 4, Thermo Fisher Scientific) for 5 min and 500 rpm. Cells were fixed with 4% PFA for 10 min at room temperature (RT) followed by a concurrent permeabilization and blocking step with 1% bovine serum albumin (BSA) (*w*/*v*), 50 µg/mL digitonin (#D141, Merck) in 1× PBS for 30 min at RT. Thereafter, the cells were incubated with the primary antibody in 1% BSA (*w*/*v*) in 1× PBS for 1 h, washed, incubated with the secondary antibody for 1 h and mounted in Fluoromount-G mounting medium (#00-4958-02, Thermo Fisher Scientific). The samples were analyzed with Zeiss LSM 710 Confocal Laser Scanning Microscopes (Carl Zeiss, Oberkochen, Germany).

### 2.8. Cell Lysis, Gel Electrophoresis and Western Blot

Cell pellets were lysed in SDS-lysis buffer (100 mM Tris-HCl pH 8, 150 mM NaCl, 10 mM EDTA, 10% SDS (*w*/*v*)), RIPA-lysis buffer (50 mM Tris-HCl pH 7.5, 150 mM NaCl, 1% TritonX-100, 0.5% sodium desoxycholate, 0.1% SDS (*w*/*v*)) or HIF-lysis buffer (6.65 M Urea, 10% Glycerol, 1% SDS (*w*/*v*), 10 mM Tris pH 6.8, adjusted to pH 7.4) freshly supplemented with protease inhibitors (cOmplete™, Mini, EDTA-free Protease Inhibitor Cocktail, Roche/Merck, #11836170001). Lysates were cleared by centrifugation. Protein concentration was measured with the Bio-Rad DC Protein assay reagent (BioRad, Dreieich, Germany, #5000111) or ROTI-Quant (Carl Roth, #K015.1), respectively. Equal protein amounts of the lysates were analyzed by SDS-PAGE and Western blot.

### 2.9. CRISPR/Cas9 and Production of Lentiviral Pseudotyped Particles

Single guide RNAs (sgRNAs) targeting human *LGMN* were designed with a CRISPR design web-interface (https://benchling.com; accessed on 13 November 2017). The human LGMN knock-out (KO) vector was obtained by cloning the annealed target-specific oligonucleotides listed in Table 2 into the BsmBI site of the pLentiCRISPRv2EGFP_ΔCas9 plasmid, a derivative of pLentiCRISPRv2 (Addgene, #52961, [36]) using the Golden Gate protocol [40]. A vector carrying a non-target control (NTC1) sequence was used as control (Table 2). After cloning, plasmids were purified and verified by sequencing. Lenti-X 293T cells (Takara Bio Europe SAS, #Z2180N) were used to produce viruses [41] using the packaging plasmids pMD2.G (Addgene, #12259) and psPAX2 (Addgene, #12260). Transduction efficiency was measured five days post transduction by flow cytometry using a BD LRS Fortessa device (Becton Dickinson, East Rutherford, NJ, USA). Data was analyzed with the BD FACSDiva software (Becton Dickinson).

### 2.10. Rescue Experiment by LGMN Wt Overexpression

RPMI-8226 target cells with stable expression of spCas9 (see Section 2.1) were cultured in RPMI medium supplemented with 10% FCS and P/S (see Section 2.1). Lentivirus production was performed in Lenti-X 293T cells (Takara, #Z2180N) cultured in DMEM (see Section 2.1). For overexpression of LGMN, the EGFP of the pHR’SIN-SEW vector [42] was first replaced with an IRES hygromycin phosphotransferase cassette to generate the initial SIHW vector. Subsequently, the cDNA of LGMN from RPMI-8226 cells was amplified with the primers CX61 (5′-GGGGGGAAGACGGGATCCACCGGTCGCCACCATGGTTTGGAAAGTAGCTGTATTC-3′) and CX62 (5′-GGGGCGTCGCGATCAGTAGTGACCAAGGCACAC-3′) and cloned with BsmBI and NruI into the vector SIHW linearized with BamHI/NruI. VSVG-pseudotyped viruses were produced as described in Section 2.9 and transduced into the RPMI-8226 lentiCas9 Blast cells. After 48h, cells were selected using hygromycin (400 ug/mL, Corning, Kaiserslautern, Germany # 30-240-CR) for additional 10 days. LGMN overexpressing cells or RPMI-8226 lentiCas9 Blast cells were subsequently transduced with NTC1 sgRNA or hLGMN-sgRNA(5) (see Table 2), which targets the translational start site of the endogenous LGMN gene but not the genetically modified overexpression construct. Cell pools with ≥98% of GFP positive cells were selected for further analysis by cumulative growth assay (Section 2.11) and Western blot (Section 2.8).

### 2.11. Quantification of Cell Proliferation: Cumulative Growth Assay and Competitive Growth Assay

Cumulative growth assays with RPMI-8226 cells were performed at a density of 4 × 10^5^ cells/mL, counted every 48 or 72 h and then reseeded to the original density in chronic hypoxia (1% O_2_) and normoxia (21% O_2_). Competitive growth assays were performed by mixing GFP-positive transduced RPMI-8226 and LP-1 cells at a ratio of (1:1) with non-transduced control cells that were monitored by flow cytometry every 48 h using a BD LRS Fortessa device (Becton Dickinson). Data were analyzed with the BD FACSDiva software (Becton Dickinson).

### 2.12. Viability Assay: IC_50_ Determination with 10t LGMN Inhibitor

IC_50_ of 10t was assessed by measuring the metabolic activity by 3-(4,5-dimethylthiazol-2-yl)-2,5-diphenyltetrazolium bromide (MTT, Sigma-Aldrich) in RPMI-8226 cells under chronic hypoxia (1% O_2_) and normoxia (21% O_2_) for 48 h. DMSO was used as a solvent control.

### 2.13. LGMN Enzyme-Linked Immunosorbent Assay (ELISA)

The Human Total legumain DuoSet ELISA (R&D Systems Inc., #DY4769) was used following the manufactuers’ instructions. Supernatants of RPMI-8226, OPM-2, U266 cells cultured under normoxic and chronic hypoxic conditions were used. LGMN standard and samples were diluted using reagent diluent (1% BSA in 1× PBS, pH 7.2–7.4, 0.2 µm filtered) and appropriate amounts of FCS (Sigma Aldrich) to receive equal FCS concentrations in standard and samples. All reagents were brought to room temperature before use. Washing was performed between the reaction steps using washing buffer (0.05% Tween 20 in 1× PBS, pH 7.2–7.4).

### 2.14. Apoptosis Assay

Cell apoptosis assay was performed by Annexin V-PE staining according to the manufacturer’s instructions (Annexin V-PE #556422, BD Biosciences, Heidelberg, Germany). Briefly, transduced RPMI-8226 spCas9 and LP1 spCas9 cells were washed twice with ice-cold PBS and cells were resuspended in 1× Annexin V Binding Buffer (10× concentrate #556454, BD Biosciences) at a concentration of 1× 10^6^ cells/mL. From this, 100 µL cell suspension were incubated with 5 µL Annexin V-PE for 30 min at 4 °C in the dark. After washing with 1× Annexin V Binding buffer, the stained cells were detected using a BD LRS Fortessa device (Becton Dickinson). Data was analyzed with the BD FACSDiva software (Becton Dickinson). As a positive control, non-transduced LP-1 spCas9 cells were incubated overnight with 500 µM H_2_O_2_ and stained with Annexin V-PE as described.

### 2.15. Statistical Analysis and Quantification

At least 3 independent experiments were performed for each assay, unless otherwise stated. For the quantification of Western blot protein bands, Image Studio 3.1 software (LI-COR Biosciences) was used. Data are shown as the mean ± S.D or as the mean ± SEM as stated in the figure legends’. The different methods for statistical comparisons were applied using GraphPad Prism 9 software and are described in the figure legends. The *p*-values were designated as follows: * *p* < 0.05; ** *p* < 0.01, *** *p* < 0.001. Values of *p* > 0.05 were considered not significant (n.s.).

## 3. Results

### 3.1. Definition of Chronic Hypoxia in MM In Vitro

**Figure 1:**
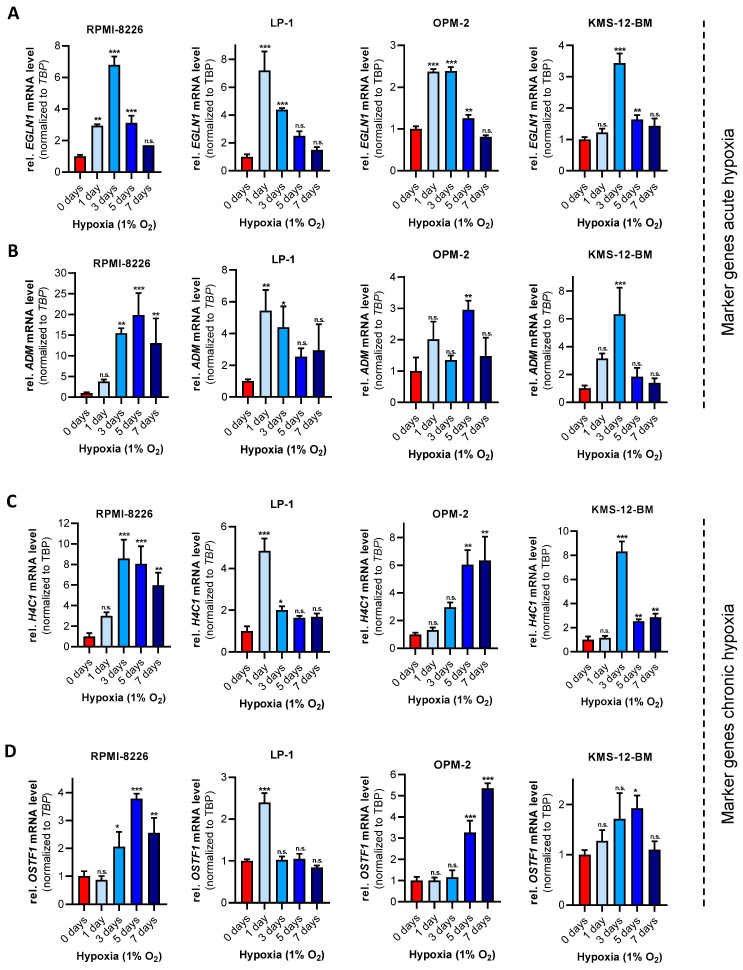
Definition of chronic hypoxia in MM cells: mRNA expression of acute and chronic hypoxia-related marker genes in RPMI-8226, LP-1, OPM-2 and KMS-12-BM cells grown at 1% O_2_ for 0, 1, 3, 5 and 7 days. (**A**) relative *EGLN1* mRNA levels normalized to *TATA-box-binding protein* (*TBP*) and to day 0. (**B**) relative *ADM* mRNA levels normalized to *TBP* and day 0. (**C**) relative *H4C1* mRNA levels normalized to *TBP* and day 0. (**D**) relative *OSTF1* mRNA levels normalized to *TBP* and day 0. Graphs indicate mRNA levels ± SD of one representative experiment, total *n* = 3. One-way ANOVA with Bonferroni’s multiple comparison test. * *p* < 0.05; ** *p* < 0.01, *** *p* < 0.001. Values of *p* > 0.05 were considered not significant (n.s.).

Within the BM microenvironment, MM cells are chronically adapted to physiologically low oxygen concentrations. Moreover, unlike solid tumor cells, liquid tumors, including MM, are able to translocate when confronted with unfavorable oxygen conditions, e.g., too low for physiological function [43]. Therefore, to analyze how chronic hypoxia affect MM cells in vitro, four MM cell lines (RPMI-8226, LP-1, OPM-2 and KMS-12-BM) were adapted to physiological oxygen concentration in vitro (1% O_2_ and 5% CO_2_) over a period of 7 days and subsequently tested for the mRNA expression of predefined marker genes for acute hypoxia (Egl-9 family hypoxia inducible factor 1, *EGLN1* (*PHD2*); adrenomedullin, *ADM*) and chronic hypoxia (H4 clustered histone 1, *H4C1*; osteoclast stimulating factor 1, *OSTF1*) [44,45] (Figure 1). For the expression of the acute hypoxia marker *EGLN1*, a significant increase was detected up to day 3, which regressed during the course of the experiment and reached the expression level of day 0 after 7 days of hypoxia (Figure 1A). A similar trend was observed for *ADM* expression in LP-1 and KMS-12-BM cells in which a significant increase was measured at days 1 and 3 or only at day 3 in KMS-12-BM (Figure 1B). For the definition of chronic hypoxia in MM, a significant increase in mRNA expression of *H4C1* and *OSTF1* was shown to be present in RPMI-8226, OPM-2, and KMS-12-BM around days 3–5. However, in the LP-1 cell line, a significant increase was measured after only one day in hypoxia (Figure 1C,D).

**Figure 2:**
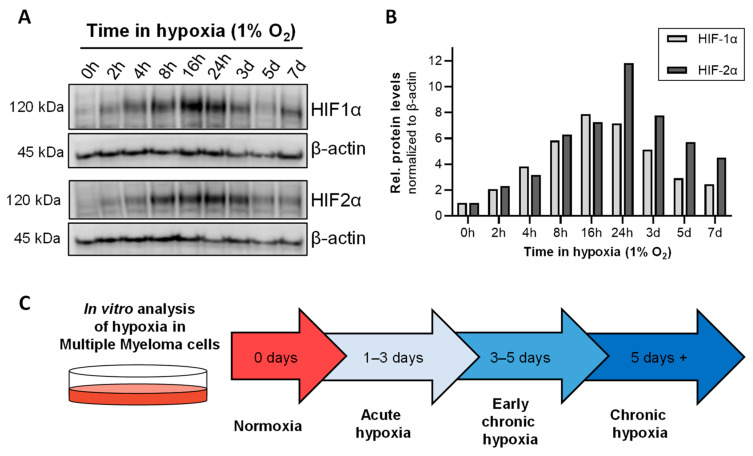
HIF1α and HIF2α levels during the adaptation to hypoxia: (**A**) Western blot of HIF1α (upper blot) and HIF2α (lower blot) levels during the adaptation to hypoxic conditions (1% O_2_) up to 7 days in RPMI-8226 cells. Loading control: β-actin. (**B**) Densitometric quantifications of protein levels (HIF1α and HIF2α). Signals were normalized to β-actin. (**C**) Model of adaptation of MM cells to hypoxia in in vitro cell culture. Abbreviations: h = hours; d = day; rel. = relative; kDa = kilodalton.

As previously mentioned, HIF1α and HIF2α are known regulators of the cellular adaptation to low oxygen levels. As shown in Figure 2A,B, the HIF1α level increases progressively after transfer of RPMI-8226 cells into hypoxia (1% O_2_) and reaches its maximum after 16 h (~7.8-fold). Thereafter, the amount of HIF1a decreases until after 7 days it is only increased ~2.5-fold compared to normoxic conditions. The kinetics of HIF2α is slightly delayed and reaches its maximum after 24 h (~11.8 fold) and also decreases to ~4.5 fold compared to normoxia.

In summary, the observations shown in Figure 1 and Figure 2 indicate that MM cells experience an acute hypoxic phase that reaches its plateau after 1–3 days. Therefore, day 1–3 at 1% O_2_ can be defined as “acute hypoxia”. Furthermore, due to the observed differences between the individual cell lines in their mRNA expression of the markers for chronic hypoxia *H4C1* and *OSTF1*, an early chronic hypoxic phase (3–5 days) and the onset of chronic hypoxia after 5–7 days can be defined (Figure 2C). Therefore, all further analyses were performed after 7 days of cell adaptation to 1% O_2_.

### 3.2. Analysis of the Global Proteome of MM Cell Lines under Chronic Hypoxic Conditions

**Figure 3:**
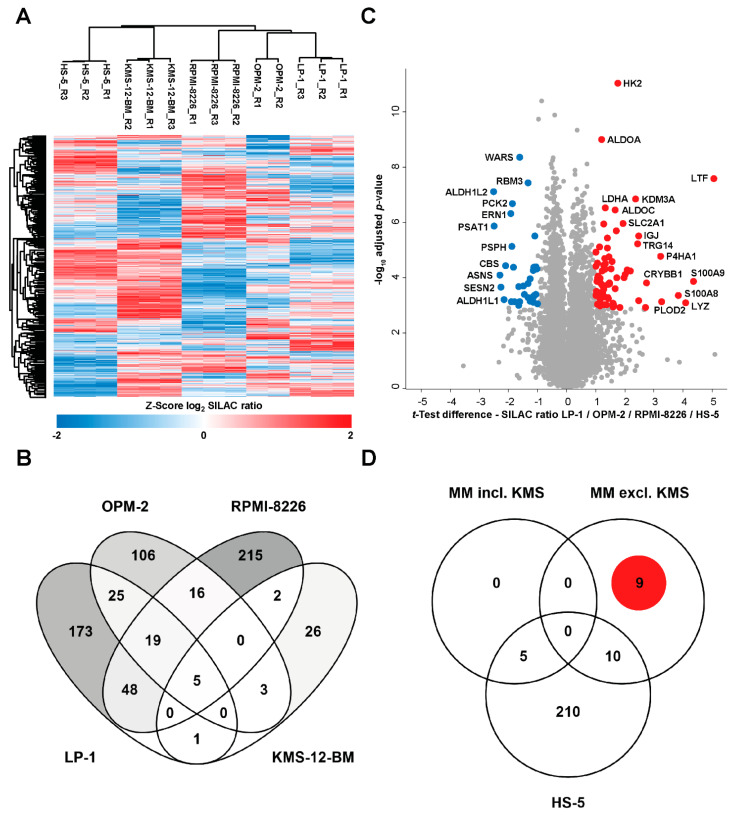
Global proteome profiling of multiple myeloma and stromal cell lines: (**A**) Heatmap of Z-transformed SILAC ratios (chronic hypoxia/normoxia) of protein groups quantified in each LC/MS analysis. Rows and columns are clustered based on the Euclidean distance and average linkage method. (**B**) Venn diagram illustrating the numbers and overlap of proteins regulated under hypoxia for the multiple myeloma cell lines under investigation. Regulated proteins were assigned by filtering for SILAC ratios showing 2-fold up- or down-regulation in at least 2 biological replicate analyses per cell line. (**C**) Volcano plot showing proteins significantly regulated under hypoxic conditions across the multiple myeloma cell lines LP-1, OPM-2, RPMI-8226 and the stromal cell line HS-5. For statistical analysis, one-sample *t*-test of the log2-transformed SILAC ratios against zero (no change) was conducted and the *p*-values were adjusted for multiple hypotheses testing (Benjamini-Hochberg FDR < 5%). (**D**) Venn diagram showing the numbers and overlap of regulated proteins between the multiple myeloma cell lines (MM) and the bone marrow stromal cell line HS-5, including and excluding MM cell line KMS-12-BM.

Subsequently, we aimed to investigate which changes in protein expression are induced by chronic hypoxia. To that end, the global proteome of MM cells (RPMI-8226, LP-1, OPM-2, KMS-12-BM) and, for comparison, the human bone marrow stromal cell line HS-5 cells chronically adapted to hypoxia (1% O_2_) for 7 days were analyzed. Overall, we quantified ~8000 protein groups across all cell lines and replicate analyses, with a median number of ca. 5500 per individual run and 3325 consistently measured in each analysis (Supplementary Tables S1 and S2). The correlation matrix of the normalized SILAC ratios from all samples showed strong correlations between biological replicates of each cell line and variable correlations between the different cell lines (Supplementary Figure S1A). The underlying protein expression differences were also reflected in an unsupervised hierarchical clustering analysis of the consistently quantified protein groups, where the biological replicates of each cell line clearly clustered together (Figure 3A). Furthermore, the protein expression heatmap showed a distinct grouping of the multiple myeloma (MM) cell lines RPMI-8226, OPM-2 and LP-1 compared to the bone marrow stromal cell line HS-5. Interestingly, the remaining MM cell line KMS-12-BM exhibited a protein expression pattern more similar to the stromal background than to the MM group, indicating a differential response to hypoxic conditions in this cell line (Figure 3, Supplementary Figure S1B). Indeed, protein expression dynamics according to the number of regulated protein groups were significantly altered in the group of RPMI-8226, OPM-2 and LP-1 compared to KMS-12-BM (Figure 3B). Thus, to focus on the characterization of hypoxia-induced alterations in MM, further analyses were conducted omitting the apparently less responsive cell line KMS-12-BM. In general, statistical analysis of SILAC ratios from hypoxia-responsive cell line samples showed a significant upregulation of proteins known to be induced under hypoxic conditions [21,46,47,48], e.g., hexokinase 2 (HK2), lactate dehydrogenase A (LDHA) and lysine demethylase 3A (KDM3A) (Figure 3C). However, a detailed comparison of regulated proteins in the responsive MM group and the stromal background revealed a subset of 9 proteins specific for the hypoxia-responsive MM cell lines (Figure 3D). These proteins showing an altered abundance upon hypoxic conditions exclusively in MM cell lines are summarized in Table 3 and include for example legumain (LGMN) and Egl-9 family hypoxia inducible factor 1 (EGLN1), better known as prolyl hydroxylase domain-containing protein 2 (PHD2).

**Figure 4:**
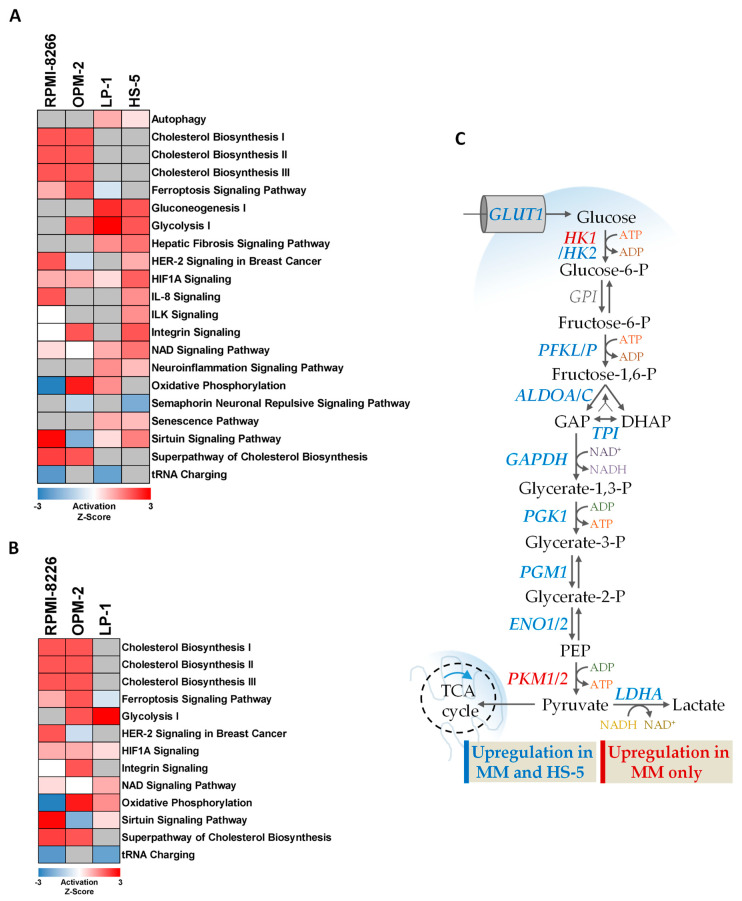
Pathway analysis of hypoxia-regulated pathways in MM and HS-5 cells: Proteins considered to be regulated in each cell line were subjected to Ingenuity core analysis and activation Z-scores of pathways scoring in at least 2 cell lines were plotted. MM cell lines RPMI-8226, LP-1 and OPM-2 and with (**A**) and without (**B**) the bone marrow stromal cell line HS-5 cells. (**C**) Illustration of the individual glycolytic enzymes identified in our LC/MS measurement. Upregulation was defined as normalized log2 SILAC ratio chronic hypoxia/normoxia >0.6. Upregulation in MM cells was defined as upregulation in at least one MM cell line. Abbreviations: P = phosphate, GAP = glyceraldehyde-3-phosphate, DHAP = dihydroxyacetone-phosphate, PEP = phosphoenolpyruvate.

To further highlight general cellular signaling pathways affected by chronic hypoxia, we conducted a pathway analysis in the three MM cell lines RPMI-8226, LP-1, and OPM-2 both with and without HS-5 cells (Figure 4). In all four cell lines we could show, that HIF1α signaling was induced in chronic hypoxia. As expected from the upregulation of the two glycolytic enzymes HK2 and LDHA, the process of glycolysis is also induced by cultivation under hypoxic conditions (OPM-2, LP-1 and HS-5) (Figure 4A,B). As shown in detail in Figure 4C, the expression of most glycolytic enzymes is induced (Supplementary Table S4). However, considering the regulation in the KMS-12-BM cells, it is noticeable that these cells do not upregulate the enzymes HK2 and LDHA (Supplementary Figure S1B). In addition, the induction of cholesterol biosynthesis in RPMI-8226 and OPM-2 cells is particularly striking. In this regard, the enzyme lanosterol 14-alpha-demethylase (CYP51A1) should be particularly mentioned, since it is significantly induced in RPMI-8226 and OPM-2 cells (Supplementary Table S1).

**Figure 5:**
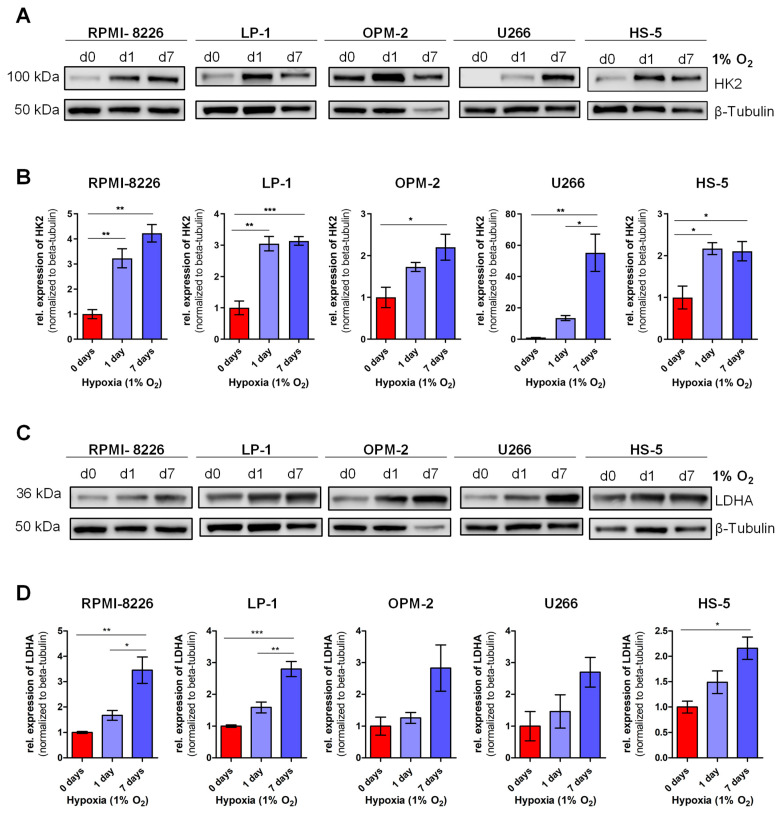
Hexokinase 2 and lactate dehydrogenase A protein expression in chronic hypoxia: Four MM cell lines and the bone marrow stromal cell line HS-5 were cultivated under hypoxic conditions and harvested at day 0, 1 and 7 for protein analysis. Representative Western blots for hexokinase 2 (HK2) (**A**) and lactate dehydrogenase A (LDHA) (**C**) are shown. Loading control: β-tubulin. Densitometric quantifications of protein expression are shown in (**B**) for HK2 and (**D**) for LDHA. Signals were normalized to β-tubulin expression. Bar graphs represent mean ±SEM of 3 independent experiments. One-way-ANOVA with Bonferroni’s post-hoc test. *, *p* < 0.05; **, *p* < 0.01; ***, *p* < 0.001. Abbreviations: d = day; rel. = relative; kDa = kilodalton; HK2 = hexokinase 2; LDHA = lactate dehydrogenase A.

For an additional validation of the results of the LC-MS/MS measurement, protein expression of HK2 and LDHA were assessed in RPMI-8226, LP-1 and OPM-2 cells and an additional MM cell line U266, as well as in the bone marrow stromal cell line HS-5 in normoxia (21% O_2_), in acute hypoxia (1% O_2_, 1 day) and chronic hypoxia (1% O_2_, 7 days). As shown in Figure 5A,B, all cell lines showed significant upregulation of HK2 protein expression from day 0 to day 7, ranging from ~2-fold upregulation in OPM-2 and HS-5 to ~60-fold upregulation in U266 cells. In addition, a significant increase in HK2 expression could be observed in acute hypoxia in RPMI-8226, LP-1 and HS-5 cells. Similarly, we showed that protein expression of LDHA in chronic hypoxia is significantly increased in RPMI-8226, LP-1 and HS-5 cells, with only a trend in OPM-2 and U266 (Figure 5C,D). In contrast, no increase in protein expression of LDHA can be detected in acute hypoxia.

In summary, our results presented in Figure 5 are consistent with the data from our LC-MS/MS analysis and suggest that, as previously described, the upregulation of glucose metabolism is a direct mechanism of adaptation to hypoxic conditions in MM and HS-5 cells.

### 3.3. MM-Specific Upregulation of the Expression of the Cysteine Protease Legumain (LGMN)

**Figure 6:**
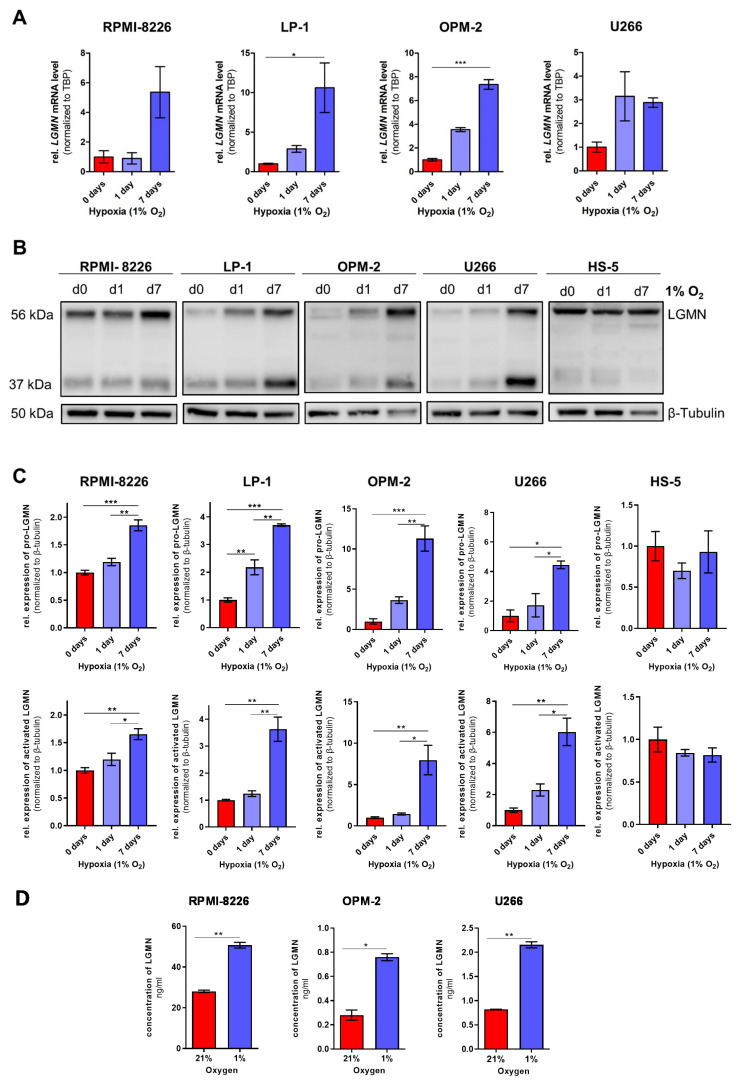
MM-specific upregulation of the cysteine protease legumain (LGMN) in chronic hypoxia: Four MM cell lines and the bone marrow stromal cell line HS-5 were cultured under hypoxic conditions (1% O_2_) and analyzed at day 0, 1 and 7 for protein and mRNA expression. (**A**) Relative *LGMN* mRNA levels in MM cell lines, normalized to *TATA-box-binding protein* (*TBP*) mRNA levels. * *p* < 0.05; ***, *p* < 0.001. (**B**) Representative Western blots for LGMN at 56 kDa (pro-LGMN) and 37 kDa (activated LGMN). β-tubulin served as a loading control. (**C**) Densitometric quantifications of LGMN expression (pro-LGMN and active LGMN). Signals were normalized to β-tubulin expression. Bar graphs represent the mean ±SEM of three independent experiments. One-way-ANOVA with Bonferroni’s post-hoc test. *, *p* < 0.05; **, *p* < 0.01; ***, *p* < 0.001. (**D**) The extracellular concentration of LGMN in RPMI-8226, OP;-2 and U266 cells was assessed by enzyme-linked immunosorbent assay (ELISA) in the supernatant under normoxic and chronic hypoxic conditions. Extracellular concentration of LGMN [ng/mL] in RPMI 8226, OPM 2 and U266 cells. Bar graphs indicate the mean ±SEM of three technical replicates. Unpaired student’s *t*-test, *, *p* < 0.05; **, *p* < 0.01.

Subsequent protein-specific analysis of the LC/MS data also revealed that, among others, the cysteine protease LGMN is strongly upregulated in chronic hypoxic MM cells (LP-1, OPM-2, and RPMI-8226) but not in HS-5 cells (Table 3). As depicted in Figure 6A, RPMI-8226, LP-1, OPM-2 and additionally U266 cells showed increasing *LGMN* mRNA expression levels over time, whereby a significant increase was observed in LP-1, and OPM-2 cells under chronic hypoxia (1% O_2_, 7 days). LGMN is synthesized as a pro-enzyme that requires the removal of the N- and C-terminal propeptides for activation [49] (Supplementary Figure S3A). The molecular weight of pro-LGMN is 56 kDa, activated LGMN can be detected at 37 kDa. As shown in Figure 6B, both the unprocessed precursor form and the active form of LGMN can be detected in MM cells, whereas HS-5 cells mainly express pro-LGMN, indicating persistent lack of activation in these cells. After 7 days of cultivation under hypoxic conditions, all MM cell lines showed a significant increase in LGMN protein expression, including both pro-LGMN and activated LGMN (Figure 6C). Although a slight increase in LGMN protein expression could already be observed in acute hypoxia (1% O_2_, 1 day, significantly only in pro-LGMN in LP-1 cells), the most significant increase in protein expression was observed on day 7. As also expected from the LC/MS data (Supplementary Tables S1 and S2), HS-5 cells showed no significant changes in LGMN expression over time. The upregulation under chronic hypoxia was also demonstrated by immunofluorescence of endogenous LGMN in LP-1, OPM2, and RPMI-8226 (Supplementary Figure S2). In both conditions, normoxia and chronic hypoxia, LGMN is mainly localized in perinuclear, punctate structures. The cytoplasmic distribution of these punctae toward the cell periphery increases under chronic hypoxia, especially in RPMI-8226 cells. In HS-5 cells, no difference in LGMN expression is observed and, moreover, no difference in cellular localization is evident.

To analyze the amount of extracellular LGMN, enzyme-linked immunosorbent assays (ELISA) were performed (Figure 6D). The ELISA assays allowed for a quantitative measurement of LGMN in the cell culture supernatants, comparing cells grown under normoxia versus chronic hypoxia. Under normoxia as well as under chronic hypoxia, LGMN was detected in the MM cells’ supernatant. Under normoxia, the RPMI 8226 cell supernatant contained more than 100 times more LGMN than the OPM-2 cell supernatant and 35 times more LGMN than U266 cells. All 3 MM cell lines exhibited a significantly increased extracellular concentration of LGMN under chronic hypoxia. Compared to normoxic conditions, the amount of LGMN in the supernatant was 1.8 to 2.6 times higher under chronic hypoxia (Figure 6D). This finding correlates with the previous results regarding the intracellular amount of pro-LGMN and active LGMN (Figure 6A–C), indicating that the intracellular amount of LGMN positively correlates with the extracellular amount of LGMN.

In summary, we demonstrated for the first time that LGMN is significantly upregulated specifically in MM cells under chronic hypoxia. In addition, under chronic hypoxia, an increased amount of LGMN is secreted into the extracellular space.

### 3.4. CRISPR/Cas9-Based Depletion of LGMN in MM Cells Confers Enhanced Growth Disadvantage under Chronic Hypoxia

**Figure 7:**
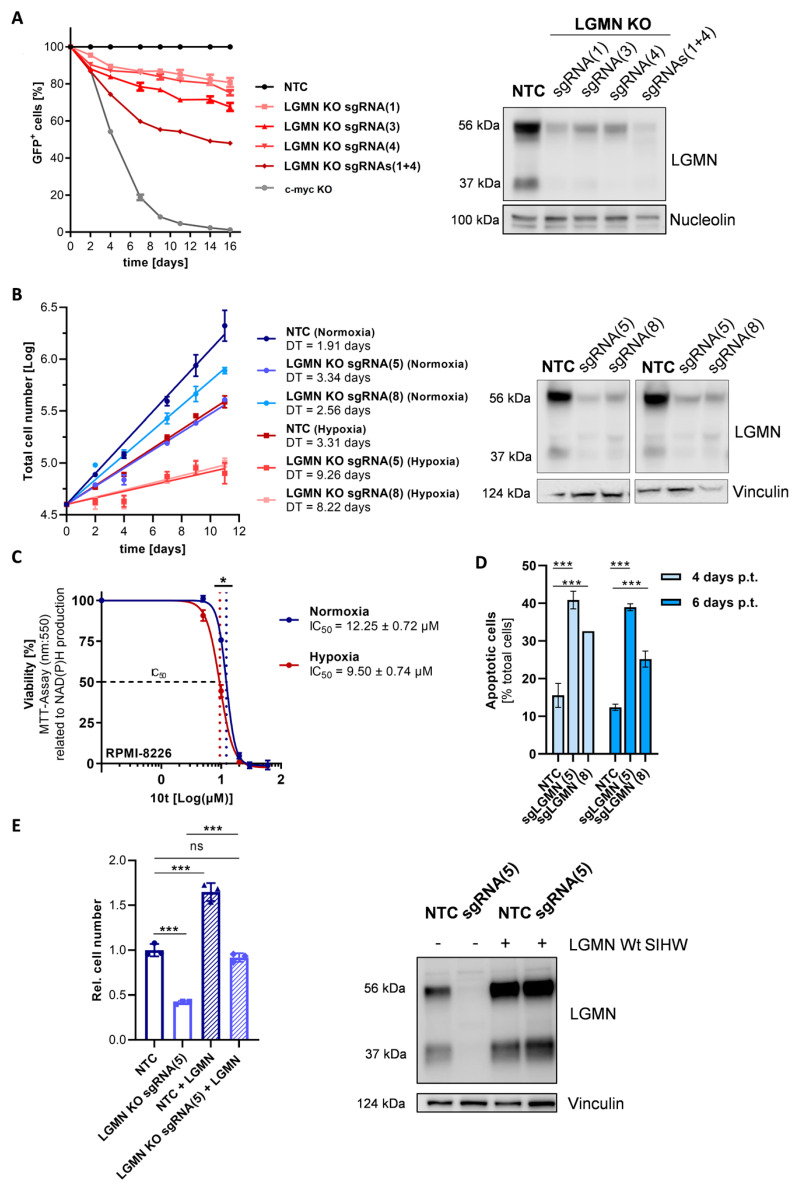
CRISPR/Cas9-based depletion of LGMN in MM cells confers enhanced growth disadvantage under chronic hypoxia: (**A**) Left: Competition growth assay of RPMI-8226 LGMN KO cells (sgRNA1, 3, 4 and 1 + 4) using NTC as a negative control and c-myc KO as a positive control in hypoxia (1% O_2_). The percentage of GFP-positive cells measured via flow cytometry is normalized to NTC and day 0. Error bars indicate mean ± SEM of three technical replicates. Right: Representative Western blot of LGMN KO in RPMI-8226 cells used in the competition assay. Loading control: Nucleolin. (**B**) Left: Cumulative growth assay of RPMI-8226 LGMN KO (sgRNA5, 8) and NTC cells in normoxia (21% O_2_, blue colors) and hypoxia (1% O_2_, red colors). Error bars indicate mean ± SEM of three technical replicates. Right: Representative Western blot of LGMN KO in RPMI-8226 cells used in the cumulative growth assay. Loading control: Vinculin. (**C**) Viability assay in RPMI-8226 cells of 3 independent experiments after treatment with LGMN inhibitor 10t for 48 h under normoxia and hypoxia. Viability in [%] measured by NAD(P)H production. IC_50_ is represented by black dashed line by blue dotted line in the graph and hypoxia by red dotted line. Error bars indicate the mean ± SEM of three biological replicates. (**D**) Apoptosis assay. Annexin V-PE-positive (apoptotic) RPMI-8226 cells [% of total cells] four and six days post transduction with sgRNA NTC, sgRNA LGMN(5) and sgRNA LGMN(8) in normoxic conditions. Bar graphs represent the mean ± SD of two independent experiments. (**E**) Rescue experiment using a pro-LGMN WT construct (LGMN Wt SIHW) in RPMI-8226 NTC and LGMN KO (sgRNA5) cells under normoxic conditions. Bar graphs represent the cumulative, relative cell number after 12 days of 3 independent experiments. Two-way-ANOVA with Bonferroni’s post-hoc test. *, *p* < 0.05; ***, *p* < 0.001. Values of *p* > 0.05 were considered not significant (ns). Abbreviations: p.t. = post transduction.

To investigate the effect of LGMN depletion on proliferation of MM cells under chronic hypoxia versus normoxia, CRISPR/Cas9-based KOs were generated using 8 distinct sgRNAs in spCas9-expressing RPMI-8226 cells (Table 2, Supplementary Figure S3). As shown in Figure 7A, depletion of LGMN results in a decreased growth rate in a competitive growth assay, with the proliferation defect becoming more pronounced with increasing knockout efficacy. A sgRNA targeting c-MYC served as a positive control. Similarly, depletion of LGMN in a cumulative growth assay leads to a significant increase in the doubling time under normoxia (~1.3–1.7 fold) as well as hypoxia (~2.5–2.8 fold), the effect being more pronounced under hypoxia (Figure 7B). Similarly, treatment with LGMN inhibitor 10t [50] leads to a decrease in cell viability under normoxia and hypoxia, with the relative IC_50_ being significantly lower under hypoxia (9.50 µM ± 0.74 µM) than under normoxia (12.25 µM ± 0.72 µM) (Figure 7C). Moreover, we showed that depletion of LGMN using two different sgRNAs resulted in a significant increase in apoptotic cells in RPMI-8226 (~13–27% increase, depending on the sgRNA) after 4 and 6 days post transduction (Figure 7D). In LP-1 cells, we observed a similar tendency (Supplementary Figure S4), yet the increase was only significant when using sgRNA LGMN(8). A difference between normoxia and chronic hypoxia is not evident (Supplementary Figure S4B) compared to proliferation (Figure 7B) and viability (Figure 7C). Finally, as shown in Figure 7E, we found that overexpression of a pro-LGMN Wt SIHW construct in RPMI-8226 cells rescues the growth inhibition after LGMN KO. In addition, overexpression of the pro-LGMN WT construct in RPMI-8226 NTC cells resulted in significantly increased total cell number.

In conclusion, we could show that depletion, as well as inhibition of LGMN, leads to a proliferation defect (Figure 7A,B) and a decrease in the cellular viability (Figure 7C), which is significantly more pronounced under hypoxia. Furthermore, independent of the oxygen level, we observe a significant increase in apoptotic cells (Figure 7D; Supplementary Figure S4).

## 4. Discussion

Hypoxia in the bone marrow microenvironment is a physiological feature of healthy and malignant cells. It has been demonstrated in vitro and in vivo that MM cells metabolically adapt to low oxygen levels and enhance molecular processes that allow them to survive and proliferate under these conditions and become more resistant to therapies [19,21,22,33,34,35]. In vitro cell culture is one of the primary tools used to study molecular mechanisms of MM pathophysiology, particularly given that primary CD138^+^ plasma cells cannot be cultured in monoculture ex vivo. Nevertheless, the environmental parameter oxygen is often neglected in mammalian cell culture [51]. While the adaptations of MM cell lines to hypoxic conditions for mostly up to 48 h have been described in a few studies [18,19,20,21,33,34,52], the adaptation to chronic hypoxia has not been explored in detail. Therefore, we first investigated the adaptation of four MM cell lines to hypoxic conditions (1% O_2_) over a period of 7 days by examining the mRNA expression of our markers for acute and chronic hypoxia, previously defined for THP-1 monocytes and macrophages [44,45]. We were able to show that the adaptation of MM cells in vitro to chronic hypoxia is established after 5–7 days (Figure 1 and Figure 2). Accordingly, the global proteome was investigated following 7 days of cultivation under hypoxic conditions (Figure 3). Pathway analysis of the global proteome under chronic hypoxia in MM and HS-5 cells revealed that cellular processes are upregulated that support adaptation to growth conditions under low oxygen levels and ensure cell survival (Figure 4). After exclusion of the data from KMS-12-BM cells due to its differential response to hypoxic conditions (Figure 3A, Supplementary Figure S1B), it is apparent that chronic hypoxia upregulates cellular HIF1α-related signaling in all four remaining cell lines. Especially HIF1α was described to suppress apoptosis in MM through the regulation of anti-apoptotic and pro-apoptotic proteins, such as myeloid cell leukemia 1 (MCL-1) [53] and BCL2 associated X (BAX))) [22,54]. In addition, inhibition of HIF1α by shRNA was shown to result in decreased tumor-induced angiogenesis and reduced tumor burden in in vivo models of MM [29]. Furthermore, HIF1 is known to play an important role in the regulation of lipid metabolism [55,56,57] and to be involved in the direct regulation of the majority of genes encoding for proteins involved in glucose uptake and glycolysis [26]. Indeed, our data show that metabolic processes such as cholesterol biosynthesis especially in RPMI-8226 and OPM-2 cells and glycolysis in OPM-2, LP-1, and HS-5 cells are also induced by chronic hypoxia (Figure 4A,B). In accordance with our data (Supplementary Table S3, Figure S4A,B), the study by Janker et al. [22] found that CD138^+^ cells derived from patients with a BM plasma cell infiltration of at least 40% upregulate proteins involved in cholesterol uptake and metabolism. Furthermore, MM patients exhibit hypocholesterinemia in the plasma, which is found to be associated with increased low-density lipoprotein (LDL) clearance and the utilization of cholesterol by MM cells [58,59,60,61,62]. Recently, in a retrospective cohort analysis, it was shown that concomitant statin therapy, which is commonly used to reduce cholesterol biosynthesis, was associated with a reduced risk of MM-specific mortality, indicating a potential role in patients with MM [63]. Moreover, we specifically identified the enzyme CYP51A1, which converts lanosterol to 4,4-dimethylcholesta-8(9),14,24-trien-3β-ol, to be significantly upregulated in chronic hypoxic MM cells (OPM-2, RPMI-8226) compared to normoxic cells (Supplementary Table S1). Taken together, this indicates that cholesterol metabolism plays an important role in the survival of MM cells. Moreover, since CYP51A1 is MM-specifically upregulated in chronic hypoxia, the development and investigation of selective CYP51A1 inhibitors may be a promising strategy for anti-myeloma treatment.

Analogous to the upregulation of cholesterol biosynthesis, we were able to show that, as previously described [21,64,65], the upregulation of glucose metabolism is a direct mechanism of adaptation to hypoxic conditions in both MM and HS-5 cells (Figure 4, Supplementary Tables S3 and S4). Specifically, regulation by HIF1α plays an important role in the upregulation of glycolysis under hypoxic conditions, as HIF1α induces the transcription of genes encoding for, e.g., HK2, aldolase A (ALDOA), LDHA, and pyruvate kinase M2 (PKM2) [66]. With respect to our data set, upregulation of glycolysis is in particular reflected by the significant upregulation of the two enzymes HK2 and LDHA in MM and HS-5 cells (Figure 3C, Figure 4C and Figure 5). Irrespective of the elevated expression levels in HS-5, MM cells in particular seem to be dependent on glucose metabolism. The study by Maiso et al. [21] demonstrated that the cultivation of MM cells for 24 h at 1% O_2_ (acute hypoxia) leads to an upregulation of the genes and metabolites of glucose metabolism. Specifically, an upregulation of *HK2* and *LDHA* expression was observed with the latter being an important target for hypoxia-driven drug resistance. Similarly, Ikeda et al. [33,67] found that the cultivation of primary MM cells derived from four distinct patients under acute hypoxia for 48 h, induced the gene expression of glycolytic enzymes, such as HK2, LDHA, and glyceraldehyde-3-phosphate dehydrogenase (GAPDH). In addition, they showed that the use of the proteasome inhibitor bortezomib in the presence of genetic inhibition of HK2 after transplantation of KMS-11 cells into immunodeficient mice or pharmacological inhibition of HK2 by 3-bromopyruvate leads to a significant tumor reduction and an increase in apoptosis induction in MM cells, respectively [33]. Another enzyme of interest is pyruvate kinase (PK), the expression of which is specifically induced in MM cells by chronic hypoxia. The PK family consists of four distinct isoforms, including the PK muscle isozyme M1 and M2. Cancer cells have been shown to predominantly express the PKM2 isoform, whereby this isoform strongly promotes the deviation of pyruvate from the TCA cycle and from OXPHOS [68]. In MM, PKM2 was shown to be induced by the c-MYC oncogene through NIMA-related kinase 2 (NEK2), and PKM2 silencing leads to a decrease in MM cell proliferation and a cell cycle arrest at G1/S transition [69,70]. Furthermore, elevated levels of PKM2 in MM patients have been proposed to indicate a poor prognosis [69,71]. In summary, already during the phase of acute hypoxia, MM and HS-5 cells increase glucose metabolism and maintain it even when chronic hypoxia is achieved. The particular dependence on MM cells, moreover, makes this metabolic process an interesting therapeutic target.

On further analysis of the individual proteins regulated by chronic hypoxia in MM and HS-5 cells, the upregulation of the histone lysine demethylase KDM3A (JHDM2A/JMJD1A) is one of the prominent findings of this proteomics study (Figure 3C). KDM3A was described to modulate histone H3 lysine 9 (H3K9) methylation for exerting transcriptional activation function [72]. Similar to our data, a HIF1α-dependent upregulation of KDM3A in response to acute hypoxia (48 h) in primary MM cells and MM cell lines was reported [48], which results in an increased expression of the long non-coding RNA metastasis associated lung adenocarcinoma transcript 1 (MALAT1). MALAT1 was previously described as a factor for poor prognosis in MM [73,74], further stabilizing HIF1α and finally its upregulation of glycolytic genes. We further identified the histone lysine demethylase KDM5C to be specifically upregulated only in MM cell lines in response to chronic hypoxia. This enzyme belongs to the KDM5 subfamily, which was found to be upregulated in prostate cancer patients [75] and to be frequently mutated in pediatric AML specimens [76] as well as in human MM cell lines [77], supporting the idea of KDM5C functioning as an oncogenic driver [78]. However, the KDM5 subfamily requires oxygen for its ability to remove di- and tri-methylations of histones H3 (H3K4). Thus, although KDM5C expression appears to be increased under chronic hypoxia, its activity appears to be decreased by oxygen deprivation, which nevertheless leads to a change in the epigenome of MM cells. Since disease progression from MGUS to MM is associated with global hypomethylation [79], it would also be worthwhile to study epigenetic changes in MM in response to chronic hypoxia in the future.

Screening for additional proteins regulated by chronic hypoxia exclusively in MM cells revealed that, the expression of the cysteine protease LGMN, also known as asparaginyl endopeptidase (AEP), is significantly induced (Table 3, Figure 6, Supplementary Figure S2). LGMN is ubiquitously expressed, yet it was reported to be increased in various types of cancers, in neurodegenerative diseases and in macrophages associated with inflammatory diseases [49,80]. Additionally, in MM, it can be deduced from the data of two studies that the gene and protein expression of LGMN is increased in primary MM cells compared to normal B cells [22,81]. As mentioned previously and indicated in Supplementary Figure S3A, LGMN is synthesized in a catalytically inactive pro-form of 56 kDa, which is autocatalytically processed into an intermediate form (46 kDa) in the acidic environment of the lysosome and then further converted to its active form of 36 kDa by other cysteine proteases. Moreover, as also suggested by our immunofluorescence images (Supplementary Figure S2), LGMN was shown to be mainly localized in the acidic environment of the lysosome, where it functions as the only known AEP, with strong preference for cleavage following asparagine residues (P1 Asn) at pH 4–5.5 and with lesser preference after aspartic acid residues (P1 Asp) at pH 4 [49,82]. As shown in Figure 6B, upregulation of LGMN by chronic hypoxia also leads to an increase in the LGMN active form, at least in LP-1, OPM-2 and U266 cells. Since overexpression of LGMN Wt SIHW also leads to re-expression of the active form and additionally to the restoration of the original proliferation rate (Figure 6D), the growth defect we observed could be due to its catalytic function as a lysosomal AEP. Moreover, normoxic and chronically hypoxic HS-5 cells almost exclusively express the pro-form and almost no active LGMN (Figure 6B), also suggesting that the processed form of LGMN, in particular, has a critical function in malignant cells.

Further review of our LGMN data reveals another interesting feature of LGMN: The link between function and localization. Similar to our finding of LGMN in the supernatant in vitro (Figure 6D), LGMN has also been detected in extracellular fluids, e.g., serum, in vivo [83]. Based on the fact that several mechanisms are active in MM cells that create a tumor-supportive environment in the BMME, such as activation of osteoclasts or suppression of osteoblasts [84,85], the increased secretion of LGMN may also play a role in the pathogenesis of MM, in general, and might contribute to the observed proliferation defect (Figure 7). As it was observed in osteoporosis, extracellular LGMN can degrade fibronectin and inhibit osteoblast differentiation, resulting in decreased bone mass [86]. Another mechanism for shaping the BMME is the secretion of exosomes, which has previously been described to be hypoxia-induced in MM [87]. Although the presence of LGMN in exosomes has not yet been studied in MM, LGMN mRNA has been described in secreted exosomes in pancreatic cancer [88] and an integrin/AEP complex in ovarian cancer [89]. For LGMN, in addition to its lysosomal localization and observed secretion, a localization in the nucleus has been described in colorectal cancer patients [90] suggesting that LGMN might work as a transcription factor as reported in plants [41]. Interestingly, closer inspection of the data of Janker et al. [22], also shows a 2.89-fold upregulation of LGMN in CD138^+^ cells compared to B-cells in the nucleus, similar to that in the cytoplasm (2.69-fold). Looking at our immunofluorescence images (Supplementary Figure S2), there also appears to be a low degree of nuclear localization, particularly in hypoxic RPMI-8226 cells.

## 5. Conclusions

In conclusion, by defining chronic hypoxia for in vitro MM monoculture, herein, we present a valuable tool to identify previously masked therapeutic vulnerabilities and to investigate oxygen-dependent physiological molecular mechanisms and metabolic dependencies in MM. The observations we presented indicate that MM cells cultured under chronic hypoxia regulate several proteins that ultimately lead directly or, in addition to their direct functions, indirectly, to an increased expression of enzymes of glycolysis. Considering the general dependence of MM cells on glucose metabolism, individual proteins identified by culturing under chronic hypoxia could be interesting targets to reduce the glycolytic activity and thus the survival of MM cells and to prevent chemoresistance, e.g., to bortezomib. Furthermore, we were able to demonstrate for the first time that the cysteine protease LGMN is induced in MM cells under chronic hypoxia. Since the depletion of LGMN leads to a significant proliferation defect and there is strong evidence that LGMN expression is also increased in primary MM cells compared to healthy cells, it would be particularly interesting to investigate in more detail the different localizations of LGMN and its associated functions in MM cells. Accordingly, we hypothesize that LGMN may act as a novel hypoxia-specific therapeutic target in MM.

## Figures and Tables

**Table 1:** Primer sequences for Real-time PCRs.

Gene	Primer Sequence 5′–3′
*EGLN1* (Egl-9 Family Hypoxia Inducible Factor 1)	GCACGACACCGGGAAGTT (forward) CCAGCTTCCCGTTACAGT (reverse)
*ADM* (Adrenomedullin)	GGATGCCGCCCGCATCCGAG (forward) GACACCAGAGTCCGACCCGG (reverse)
*H4C1* (H4 Clustered Histone 1)	AAGCGCATTTCTGGTCTCAT (forward) AAGGGCCTTTTGGAGTCTGT (reverse)
*OSTF1* (Osteoclast Stimulating Factor 1)	TTGCATGAAGCAGCAAAAAG (forward) TAAGGCAGTGCTTCCAGCTT (reverse)
*TBP* (TATA-box-binding protein)	GGAATCCCTATCTTTAGTCCAAT (forward) GACTATTGGTGTTCTGAATAGGC (reverse)
*LGMN* (Legumain)	ATCCTGAAGATGGAGGCAAG (forward) TTGCGGTGAATGATCTGGTA (reverse)

**Table 2:** Oligonucleotides for sgRNAs.

Gene	Primer Sequence 5′–3′
h*LGMN*-sgRNA1	CACCGgcgatgcagaagcagtgaa (forward) AAACttcactgcttctgcatcgcC (reverse)
h*LGMN*-sgRNA2	CACCGttgtgatcaacaggcccaa (forward) AAACttgggcctgttgatcacaaC (reverse)
h*LGMN*-sgRNA3	CACCGttcgtcaggaatcccattg (forward) AAACcaatgggattcctgacgaaC (reverse)
h*LGMN*-sgRNA4	CACCGttgcggtgaatgatctggt (forward) AAACaccagatcattcaccgcaaC (reverse)
h*LGMN*-sgRNA5	CACCGtccaaggtgcagaatggtt (forward) AAACaaccattctgcaccttggaC (reverse)
h*LGMN*-sgRNA6	CACCGctggactcctccagatcat (forward) AAACatgatctggaggagtccagC (reverse)
h*LGMN*-sgRNA7	CACCGaaatggctggtataattat (forward) AAACataattataccagccatttC (reverse)
h*LGMN*-sgRNA8	CACCGgccccgtctgcctcacaga (forward) AAACtctgtgaggcagacggggcC (reverse)
h*MYC*	CACCGtctgagacgagcttggcgg (forward) AAACccgccaagctcgtctcagaC (reverse)
NTC1	CACCGttccgggctaacaagtcct (forward) AAACaggacttgttagcccggaaC (reverse)

**Table 3:** Significant hypoxia-regulated proteins in Multiple Myeloma cell lines LP-1, OPM-2 and RPMI-8226.

Protein Name	Median log2 SILAC Ratio Chronic Hypoxia/Normoxia			Peptides	Unique Peptides	Sequence Coverage [%]
	LP-1	OPM-2	RPMI-8226			
JCHAIN	2.9	2.7	1.9	11	11	67
LGMN	2.2	2.7	1.4	18	15	59
EGLN1	1.5	1.7	1.2	14	1	43
HMOX1	−1.6	1.7	2.4	9	9	37
RRM1	1.3	1.5	3.3	37	37	61
KDM5C	2.0	1.4	2.2	53	1	46
SARS1	−1.3	−1.1	−1.4	32	32	56
ISOC1	−1.0	−1.4	−1.3	14	14	65
PSPH	−2.0	−1.6	−2.7	19	19	73

## Data Availability

The mass spectrometry proteomics data have been deposited to the Proteo-meXchange Consortium via the PRIDE [37] partner repository with the dataset identifier PXD030239.

## Supplementary Materials

The following supporting information can be downloaded at: https://www.mdpi.com/article/10.3390/cells11020292/s1, Supplementary Table S1: LC/MS data; Supplementary Table S2: *t*-test data LC/MS data; Supplementary Table S3: Ingenuity pathway analysis; Supplementary Table S4: LC/MS data—regulation of glycolytic enzymes; Supplementary Figure S1: Correlation of global proteome profiles and significantly regulated proteins in KMS-12-BM cells: (A) The matrix was created by plotting the Pearson correlation coefficient based on the SILAC ratios of each quantified protein in individual LC/MS analyses against all other runs. (B) Volcano plot showing proteins significantly regulated under hypoxic conditions KMS-12-BM cells. For statistical analysis, one-sample *t*-test of the log2-transformed SILAC ratios against zero (no change) was conducted and the *p*-values were adjusted for multiple hypotheses testing (Benjamini-Hochberg FDR < 5%); Supplementary Figure S2: Confocal images of the Multiple Myeloma-specific upregulation of LGMN in chronic hypoxia: Three MM cell lines and the stromal cell line HS-5 were cultured under hypoxic conditions (1% O_2_) for 7 days and stained for endogenous LGMN. Nuclei were visualized using DAPI. Scale bar = 20 µM; Supplementary Figure S3: Knockout of LGMN in RPMI-8226 cells using eight distinct sgRNAs: (A) Schematic view of the gene structure of LGMN. The domains of LGMN protein, and the complementary regions of the sgRNAs used, are indicated. SP, signal peptide; NPP, N-terminal propeptide, AP, activation peptide, LSAM, LGMN stabilization and activity modulation domain (C-terminal propeptide). (B) Knockout of LGMN in RPMI-8226 cells using eight distinct sgRNAs. Representative Westernblot of LGMN. Loading control: Nucleolin; Supplementary Figure S4: Knockout of LGMN in LP-1 cells leads to an oxygen-independent induction of apoptosis: (A) Annexin V-PE-positive (apoptotic) LP-1 cells [% of total cells] four and six days post transduction with sgRNA NTC, sgRNA LGMN(5) and sgRNA LGMN(8) in normoxic conditions. Bar graphs represent the mean ± SD of two independent experiments. Two-way-ANOVA with Bonferroni’s post hoc test. *, *p* < 0.05. Abbreviations: p.t. = post transduction. (B) Annexin V-PE-positive (apoptotic) LP-1 cells [% of total cells] four days post transduction with sgRNA NTC, sgRNA LGMN(5) and sgRNA LGMN(8) in normoxic and chronic hypoxic conditions. Bar graphs represent the mean ± SD of two independent experiments. Two-way-ANOVA with Bonferroni’s post-hoc test. *, *p* < 0.05. Data under normoxia are the same as shown in (A) for four days post transduction.

## References

[1] Cowan A.J., Allen C., Barac A., Basaleem H., Bensenor I., Curado M.P., Foreman K., Gupta R., Harvey J., Hosgood H.D. (2018). Global Burden of Multiple Myeloma: A Systematic Analysis for the Global Burden of Disease Study 2016. JAMA Oncol..

[2] Ludwig H., Novis Durie S., Meckl A., Hinke A., Durie B. (2020). Multiple Myeloma Incidence and Mortality Around the Globe; Interrelations Between Health Access and Quality, Economic Resources, and Patient Empowerment. Oncologist.

[3] Dingli D., Ailawadhi S., Bergsagel P.L., Buadi F.K., Dispenzieri A., Fonseca R., Gertz M.A., Gonsalves W.I., Hayman S.R., Kapoor P. (2017). Therapy for Relapsed Multiple Myeloma: Guidelines from the Mayo Stratification for Myeloma and Risk-Adapted Therapy. Mayo Clin. Proc..

[4] Joshua D.E., Bryant C., Dix C., Gibson J., Ho J. (2019). Biology and therapy of multiple myeloma. Med. J. Aust..

[5] Manier S., Sacco A., Leleu X., Ghobrial I.M., Roccaro A.M. (2012). Bone marrow microenvironment in multiple myeloma progression. J. Biomed. Biotechnol..

[6] Hou J., Wei R., Qian J., Wang R., Fan Z., Gu C., Yang Y. (2019). The impact of the bone marrow microenvironment on multiple myeloma (Review). Oncol. Rep..

[7] García-Ortiz A., Rodríguez-García Y., Encinas J., Maroto-Martín E., Castellano E., Teixidó J., Martínez-López J. (2021). The Role of Tumor Microenvironment in Multiple Myeloma Development and Progression. Cancers.

[8] Faict S., Muller J., De Veirman K., De Bruyne E., Maes K., Vrancken L., Heusschen R., De Raeve H., Schots R., Vanderkerken K. (2018). Exosomes play a role in multiple myeloma bone disease and tumor development by targeting osteoclasts and osteoblasts. Blood Cancer J..

[9] Vacca A., Ria R., Semeraro F., Merchionne F., Coluccia M., Boccarelli A., Scavelli C., Nico B., Gernone A., Battelli F. (2003). Endothelial cells in the bone marrow of patients with multiple myeloma. Blood.

[10] Ribatti D., Vacca A. (2016). Role of Endothelial Cells and Fibroblasts in Multiple Myeloma Angiogenic Switch. Cancer Treat Res..

[11] Nombela-Arrieta C., Pivarnik G., Winkel B., Canty K.J., Harley B., Mahoney J.E., Park S.Y., Lu J., Protopopov A., Silberstein L.E. (2013). Quantitative imaging of haematopoietic stem and progenitor cell localization and hypoxic status in the bone marrow microenvironment. Nat. Cell Biol..

[12] Spencer J.A., Ferraro F., Roussakis E., Klein A., Wu J., Runnels J.M., Zaher W., Mortensen L.J., Alt C., Turcotte R. (2014). Direct measurement of local oxygen concentration in the bone marrow of live animals. Nature.

[13] Suda T., Takubo K., Semenza G.L. (2011). Metabolic Regulation of Hematopoietic Stem Cells in the Hypoxic Niche. Cell Stem Cell.

[14] Mantel C.R., O’Leary H., Chitteti B.R., Huang X., Cooper S., Hangoc G., Brustovetsky N., Srour E.F., Lee M.R., Messina-Graham S. (2015). Enhancing Hematopoietic Stem Cell Transplantation Efficacy by Mitigating Oxygen Shock. Cell.

[15] van Oosterwijk J., Buelow D.R., Drenberg C.D., Vasilyeva A., Li L., Shi L., Wang Y.-D., Finkelstein D., Shurtleff S.A., Janke L.J. (2017). Hypoxia-induced upregulation of BMX kinase mediates therapeutic resistance in acute myeloid leukemia. J. Clin. Investig..

[16] Baccelli I., Gareau Y., Lehnertz B., Gingras S., Spinella J.-F., Corneau S., Mayotte N., Girard S., Frechette M., Blouin-Chagnon V. (2019). Mubritinib Targets the Electron Transport Chain Complex I and Reveals the Landscape of OXPHOS Dependency in Acute Myeloid Leukemia. Cancer Cell.

[17] Colla S., Storti P., Donofrio G., Todoerti K., Bolzoni M., Lazzaretti M., Abeltino M., Ippolito L., Neri A., Ribatti D. (2010). Low bone marrow oxygen tension and hypoxia-inducible factor-1α overexpression characterize patients with multiple myeloma: Role on the transcriptional and proangiogenic profiles of CD138+ cells. Leukemia.

[18] Azab A.K., Hu J., Quang P., Azab F., Pitsillides C., Awwad R., Thompson B., Maiso P., Sun J.D., Hart C.P. (2012). Hypoxia promotes dissemination of multiple myeloma through acquisition of epithelial to mesenchymal transition-like features. Blood.

[19] Kawano Y., Kikukawa Y., Fujiwara S., Wada N., Okuno Y., Mitsuya H., Hata H. (2013). Hypoxia reduces CD138 expression and induces an immature and stem cell-like transcriptional program in myeloma cells. Int. J. Oncol..

[20] Muz B., De La Puente P., Azab F., Luderer M., Azab A.K. (2014). Hypoxia promotes stem cell-like phenotype in multiple myeloma cells. Blood Cancer J..

[21] Maiso P., Huynh D., Moschetta M., Sacco A., Aljawai Y., Mishima Y., Asara J.M., Roccaro A.M., Kimmelman A.C., Ghobrial I.M. (2015). Metabolic Signature Identifies Novel Targets for Drug Resistance in Multiple Myeloma. Cancer Res..

[22] Janker L., Mayer R.L., Bileck A., Kreutz D., Mader J.C., Utpatel K., Heudobler D., Agis H., Gerner C., Slany A. (2019). Metabolic, Anti-apoptotic and Immune Evasion Strategies of Primary Human Myeloma Cells Indicate Adaptations to Hypoxia*. Mol. Cell. Proteom..

[23] Semenza G.L., Wang G.L. (1992). A nuclear factor induced by hypoxia via de novo protein synthesis binds to the human erythropoietin gene enhancer at a site required for transcriptional activation. Mol. Cell Biol..

[24] Wenger R.H., Stiehl D.P., Camenisch G. (2005). Integration of oxygen signaling at the consensus HRE. Sci. STKE.

[25] Ortiz-Barahona A., Villar D., Pescador N., Amigo J., Del Peso L. (2010). Genome-wide identification of hypoxia-inducible factor binding sites and target genes by a probabilistic model integrating transcription-profiling data and in silico binding site prediction. Nucleic Acids Res..

[26] Denko N.C. (2008). Hypoxia, HIF1 and glucose metabolism in the solid tumour. Nat. Rev. Cancer.

[27] Martin S.K., Diamond P., Williams S.A., To L.B., Peet D.J., Fujii N., Gronthos S., Harris A.L., Zannettino A.C. (2010). Hypoxia-inducible factor-2 is a novel regulator of aberrant CXCL12 expression in multiple myeloma plasma cells. Haematologica.

[28] Hu Y., Kirito K., Yoshida K., Mitsumori T., Nakajima K., Nozaki Y., Hamanaka S., Nagashima T., Kunitama M., Sakoe K. (2009). Inhibition of hypoxia-inducible factor-1 function enhances the sensitivity of multiple myeloma cells to melphalan. Mol. Cancer Ther..

[29] Storti P., Bolzoni M., Donofrio G., Airoldi I., Guasco D., Toscani D., Martella E., Lazzaretti M., Mancini C., Agnelli L. (2013). Hypoxia-inducible factor (HIF)-1α suppression in myeloma cells blocks tumoral growth in vivo inhibiting angiogenesis and bone destruction. Leukemia.

[30] Borsi E., Perrone G., Terragna C., Martello M., Dico A.F., Solaini G., Baracca A., Sgarbi G., Pasquinelli G., Valente S. (2014). Hypoxia inducible factor-1 alpha as a therapeutic target in multiple myeloma. Oncotarget.

[31] Kocemba-Pilarczyk K.A., Ostrowska B., Trojan S., Aslan E., Kusior D., Lasota M., Lenouvel C., Dulinska-Litewka J. (2018). Targeting the hypoxia pathway in malignant plasma cells by using 17-allylamino-17-demethoxygeldanamycin. Acta Biochim. Pol..

[32] Kocemba-Pilarczyk K.A., Trojan S., Ostrowska B., Lasota M., Dudzik P., Kusior D., Kot M. (2020). Influence of metformin on HIF-1 pathway in multiple myeloma. Pharmacol. Rep..

[33] Ikeda S., Abe F., Matsuda Y., Kitadate A., Takahashi N., Tagawa H. (2020). Hypoxia-inducible hexokinase-2 enhances anti-apoptotic function via activating autophagy in multiple myeloma. Cancer Sci..

[34] Nakagawa Y., Ashihara E., Yao H., Yokota A., Toda Y., Miura Y., Nakata S., Hirai H., Maekawa T. (2018). Multiple myeloma cells adapted to long-exposure of hypoxia exhibit stem cell characters with TGF-beta/Smad pathway activation. Biochem. Biophys. Res. Commun..

[35] Silva A.S., Gatenby R.A. (2011). Adaptation to Survival in Germinal Center Is the Initial Step in Onset of Indolent Stage of Multiple Myeloma. Mol. Pharm..

[36] Sanjana N.E., Shalem O., Zhang F. (2014). Improved vectors and genome-wide libraries for CRISPR screening. Nat. Methods.

[37] Perez-Riverol Y., Csordas A., Bai J., Bernal-Llinares M., Hewapathirana S., Kundu D.J., Inuganti A., Griss J., Mayer G., Eisenacher M. (2019). The PRIDE database and related tools and resources in 2019: Improving support for quantification data. Nucleic Acids Res..

[38] Cox J., Neuhauser N., Michalski A., Scheltema R.A., Olsen J.V., Mann M. (2011). Andromeda: A peptide search engine integrated into the MaxQuant environment. J. Proteome Res..

[39] Tyanova S., Temu T., Sinitcyn P., Carlson A., Hein M.Y., Geiger T., Mann M., Cox J. (2016). The Perseus computational platform for comprehensive analysis of (prote)omics data. Nat. Methods.

[40] Engler C., Kandzia R., Marillonnet S. (2008). A one pot, one step, precision cloning method with high throughput capability. PLoS ONE.

[41] Tiscornia G., Singer O., Verma I.M. (2006). Production and purification of lentiviral vectors. Nat. Protoc..

[42] Demaison C., Parsley K., Brouns G., Scherr M., Battmer K., Kinnon C., Grez M., Thrasher A.J. (2002). High-level transduction and gene expression in hematopoietic repopulating cells using a human immunodeficiency [correction of imunodeficiency] virus type 1-based lentiviral vector containing an internal spleen focus forming virus promoter. Hum. Gene Ther..

[43] Rieger C.T., Fiegl M. (2016). Microenvironmental oxygen partial pressure in acute myeloid leukemia: Is there really a role for hypoxia?. Exp. Hematol..

[44] Fuhrmann D.C., Wittig I., Heide H., Dehne N., Brune B. (2013). Chronic hypoxia alters mitochondrial composition in human macrophages. Biochim. Biophys. Acta.

[45] Fuhrmann D.C., Tausendschön M., Wittig I., Steger M., Ding M.G., Schmid T., Dehne N., Brüne B. (2015). Inactivation of Tristetraprolin in Chronic Hypoxia Provokes the Expression of Cathepsin B. Mol. Cell. Biol..

[46] Uemura M., Yamamoto H., Takemasa I., Mimori K., Hemmi H., Mizushima T., Ikeda M., Sekimoto M., Matsuura N., Doki Y. (2010). Jumonji Domain Containing 1A Is a Novel Prognostic Marker for Colorectal Cancer: In vivo Identification from Hypoxic Tumor Cells. Clin. Cancer Res..

[47] Wade M., Jones D., Wilson L., Stockley J., Coffey K., Robson C.N., Gaughan L. (2014). The histone demethylase enzyme KDM3A is a key estrogen receptor regulator in breast cancer. Nucleic Acids Res..

[48] Ikeda S., Kitadate A., Abe F., Takahashi N., Tagawa H. (2018). Hypoxia-inducible KDM3A addiction in multiple myeloma. Blood Adv..

[49] Dall E., Brandstetter H. (2016). Structure and function of legumain in health and disease. Biochimie.

[50] Ness K.A., Eddie S.L., Higgins C.A., Templeman A., D’Costa Z., Gaddale K.K., Bouzzaoui S., Jordan L., Janssen D., Harrison T. (2015). Development of a potent and selective cell penetrant Legumain inhibitor. Bioorg. Med. Chem. Lett..

[51] Ast T., Mootha V.K. (2019). Oxygen and mammalian cell culture: Are we repeating the experiment of Dr. Ox?. Nat. Metab..

[52] Martin T., Baz R., Benson D.M., Lendvai N., Wolf J., Munster P., Lesokhin A.M., Wack C., Charpentier E., Campana F. (2017). A phase 1b study of isatuximab plus lenalidomide and dexamethasone for relapsed/refractory multiple myeloma. Blood.

[53] Wu F., Tong D.D., Ni L., Wang L.M., Wang M.C. (2020). HIF-1alpha suppresses myeloma progression by targeting Mcl-1. Int. J. Clin. Exp. Pathol..

[54] Borsi E., Terragna C., Brioli A., Tacchetti P., Martello M., Cavo M. (2014). Therapeutic targeting of hypoxia and hypoxia-inducible factor 1 alpha in multiple myeloma. Transl. Res..

[55] Valli A., Rodriguez M., Moutsianas L., Fischer R., Fedele V., Huang H.L., Van Stiphout R., Jones D., McCarthy M., Vinaxia M. (2015). Hypoxia induces a lipogenic cancer cell phenotype via HIF1alpha-dependent and -independent pathways. Oncotarget.

[56] Shen G.-M., Zhao Y.-Z., Chen M.-T., Zhang F.-L., Liu X.-L., Wang Y., Liu C.-Z., Yu J., Zhang J.-W. (2011). Hypoxia-inducible factor-1 (HIF-1) promotes LDL and VLDL uptake through inducing VLDLR under hypoxia. Biochem. J..

[57] Shen G., Li X., Zheng J., Zhou C. (2017). The Multifaceted Role of Hypoxia-Inducible Factor 1 (HIF1) in Lipid Metabolism. Lipid Metabolism, Hypoxia and Human Diseases.

[58] Tirado-Vélez J.M., Benítez-Rondán A., Cózar-Castellano I., Medina F., Perdomo G. (2011). Low-density lipoprotein cholesterol suppresses apoptosis in human multiple myeloma cells. Ann. Hematol..

[59] Yavasoglu I., Tombuloglu M., Kadikoylu G., Donmez A., Cagirgan S., Bolaman Z., Cagırgan S. (2007). Cholesterol levels in patients with multiple myeloma. Ann. Hematol..

[60] Liu X., Xu P., Wang L., Zhang C., Wang M., Ouyang J., Chen B. (2020). Cholesterol Levels Provide Prognostic Information in Patients with Multiple Myeloma. Clin. Lab..

[61] Liang L., Li J., Fu H., Liu X., Liu P. (2019). Identification of High Serum Apolipoprotein A1 as a Favorable Prognostic Indicator in Patients with Multiple Myeloma. J. Cancer.

[62] Lazaris V., Hatziri A., Symeonidis A., Kypreos K.E. (2021). The Lipoprotein Transport System in the Pathogenesis of Multiple Myeloma: Advances and Challenges. Front. Oncol..

[63] Sanfilippo K.M., Keller J., Gage B.F., Luo S., Wang T.-F., Moskowitz G., Gumbel J., Blue B., O’Brian K., Carson K.R. (2016). Statins Are Associated with Reduced Mortality in Multiple Myeloma. J. Clin. Oncol..

[64] Eales K.L., Hollinshead K.E.R., Tennant D.A. (2016). Hypoxia and metabolic adaptation of cancer cells. Oncogenesis.

[65] Al Tameemi W., Dale T.P., Al-Jumaily R.M.K., Forsyth N.R. (2019). Hypoxia-Modified Cancer Cell Metabolism. Front. Cell Dev. Biol..

[66] Xia X., Lemieux M.E., Li W., Carroll J.S., Brown M., Liu X.S., Kung A.L. (2009). Integrative analysis of HIF binding and transactivation reveals its role in maintaining histone methylation homeostasis. Proc. Natl. Acad. Sci. USA.

[67] Ikeda S., Kitadate A., Abe F., Saitoh H., Michishita Y., Hatano Y., Kawabata Y., Kitabayashi A., Teshima K., Kume M. (2017). Hypoxia-inducible microRNA-210 regulates the DIMT1-IRF4 oncogenic axis in multiple myeloma. Cancer Sci..

[68] Christofk H.R., Vander Heiden M.G., Harris M.H., Ramanathan A., Gerszten R.E., Wei R., Fleming M.D., Schreiber S.L., Cantley L.C. (2008). The M2 splice isoform of pyruvate kinase is important for cancer metabolism and tumour growth. Nature.

[69] Gu Z., Xia J., Xu H., Frech I., Tricot G., Zhan F. (2017). NEK2 Promotes Aerobic Glycolysis in Multiple Myeloma Through Regulating Splicing of Pyruvate Kinase. J. Hematol. Oncol..

[70] He Y., Wang Y., Liu H., Xu X., He S., Tang J., Huang Y., Miao X., Wu Y., Wang Q. (2015). Pyruvate kinase isoform M2 (PKM2) participates in multiple myeloma cell proliferation, adhesion and chemoresistance. Leuk. Res..

[71] Panchabhai S., Schlam I., Sebastian S., Fonseca R. (2016). PKM2 and other key regulators of Warburg effect positively correlate with CD147 (EMMPRIN) gene expression and predict survival in multiple myeloma. Leukemia.

[72] Yamane K., Toumazou C., Tsukada Y.-I., Erdjument-Bromage H., Tempst P., Wong J., Zhang Y. (2006). JHDM2A, a JmjC-Containing H3K9 Demethylase, Facilitates Transcription Activation by Androgen Receptor. Cell.

[73] Cho S.F., Chang Y.C., Chang C.S., Lin S.F., Liu Y.C., Hsiao H.H., Chang J.G., Liu T.C. (2014). MALAT1 long non-coding RNA is overexpressed in multiple myeloma and may serve as a marker to predict disease progression. BMC Cancer.

[74] Handa H., Kuroda Y., Kimura K., Masuda Y., Hattori H., Alkebsi L., Matsumoto M., Kasamatsu T., Kobayashi N., Tahara K.I. (2017). Long non-coding RNA MALAT1 is an inducible stress response gene associated with extramedullary spread and poor prognosis of multiple myeloma. Br. J. Haematol..

[75] Stein J., Majores M., Rohde M., Lim S., Schneider S., Krappe E., Ellinger J., Dietel M., Stephan C., Jung K. (2014). KDM5C Is Overexpressed in Prostate Cancer and Is a Prognostic Marker for Prostate-Specific Antigen-Relapse Following Radical Prostatectomy. Am. J. Pathol..

[76] Zhan D., Zhang Y., Xiao P., Zheng X., Ruan M., Zhang J., Chen A., Zou Y., Chen Y., Huang G. (2018). Whole exome sequencing identifies novel mutations of epigenetic regulators in chemorefractory pediatric acute myeloid leukemia. Leuk. Res..

[77] Vikova V., Jourdan M., Robert N., Requirand G., Boireau S., Bruyer A., Vincent L., Cartron G., Klein B., Elemento O. (2019). Comprehensive characterization of the mutational landscape in multiple myeloma cell lines reveals potential drivers and pathways associated with tumor progression and drug resistance. Theranostics.

[78] Plch J., Hrabeta J., Eckschlager T. (2018). KDM5 demethylases and their role in cancer cell chemoresistance. Int. J. Cancer.

[79] Walker B.A., Wardell C., Chiecchio L., Smith E.M., Boyd K., Neri A., Davies F.E., Ross F.M., Morgan G. (2011). Aberrant global methylation patterns affect the molecular pathogenesis and prognosis of multiple myeloma. Blood.

[80] Zhen Y., Chunlei G., Wenzhi S., Shuangtao Z., Na L., Rongrong W., Xiaohe L., Haiying N., Dehong L., Shan J. (2015). Clinicopathologic significance of legumain overexpression in cancer: A systematic review and meta-analysis. Sci. Rep..

[81] De Vos J., Thykjaer T., Tarte K., Ensslen M., Raynaud P., Requirand G., Pellet F., Pantesco V., Rème T., Jourdan M. (2002). Comparison of gene expression profiling between malignant and normal plasma cells with oligonucleotide arrays. Oncogene.

[82] Poreba M. (2019). Recent advances in the development of legumain-selective chemical probes and peptide prodrugs. Biol. Chem..

[83] Umei T.C., Kishimoto Y., Aoyama M., Saita E., Niki H., Ikegami Y., Ohmori R., Kondo K., Momiyama Y. (2020). High Plasma Levels of Legumain in Patients with Complex Coronary Lesions. J. Atheroscler. Thromb..

[84] Tian E., Zhan F., Walker R., Rasmussen E., Ma Y., Barlogie B., Shaughnessy J.D. (2003). The Role of the Wnt-Signaling Antagonist DKK1 in the Development of Osteolytic Lesions in Multiple Myeloma. N. Engl. J. Med..

[85] Kawano Y., Moschetta M., Manier S., Glavey S., Görgün G.T., Roccaro A.M., Anderson K.C., Ghobrial I.M. (2015). Targeting the bone marrow microenvironment in multiple myeloma. Immunol. Rev..

[86] Jafari A., Qanie D., Andersen T.L., Zhang Y., Chen L., Postert B., Parsons S., Ditzel N., Khosla S., Johansen H.T. (2017). Legumain Regulates Differentiation Fate of Human Bone Marrow Stromal Cells and Is Altered in Postmenopausal Osteoporosis. Stem Cell Rep..

[87] Umezu T., Tadokoro H., Azuma K., Yoshizawa S., Ohyashiki K., Ohyashiki J.H. (2014). Exosomal miR-135b shed from hypoxic multiple myeloma cells enhances angiogenesis by targeting factor-inhibiting HIF-1. Blood.

[88] Chen J., Wang S., Jia S., Ding G., Jiang G., Cao L. (2018). Integrated Analysis of Long Non-Coding RNA and mRNA Expression Profile in Pancreatic Cancer Derived Exosomes Treated Dendritic Cells by Microarray Analysis. J. Cancer.

[89] Li X., Tang M., Zhu Q., Wang X., Lin Y., Wang X. (2020). The exosomal integrin alpha5beta1/AEP complex derived from epithelial ovarian cancer cells promotes peritoneal metastasis through regulating mesothelial cell proliferation and migration. Cell Oncol..

[90] Haugen M.H., Boye K., Nesland J.M., Pettersen S.J., Egeland E.V., Tamhane T., Brix K., Maelandsmo G.M., Flatmark K. (2015). High expression of the cysteine proteinase legumain in colorectal cancer—Implications for therapeutic targeting. Eur. J. Cancer.

