# Supplementary material for: Identification of the Cysteine Protease Legumain as a Potential Chronic Hypoxia-Specific Multiple Myeloma Target Gene
Source: Cells. 2022 Jan 15;11(2):292. doi: 10.3390/cells11020292 (PMC8773999; doi:10.3390/cells11020292)
Supplement: Supplementary file 1 [file cells-11-00292-s001.zip › cells-1465172 - supplementary/supplementary-figures and tables/Figure S1-S4.pdf]

Supplementary Figure S1

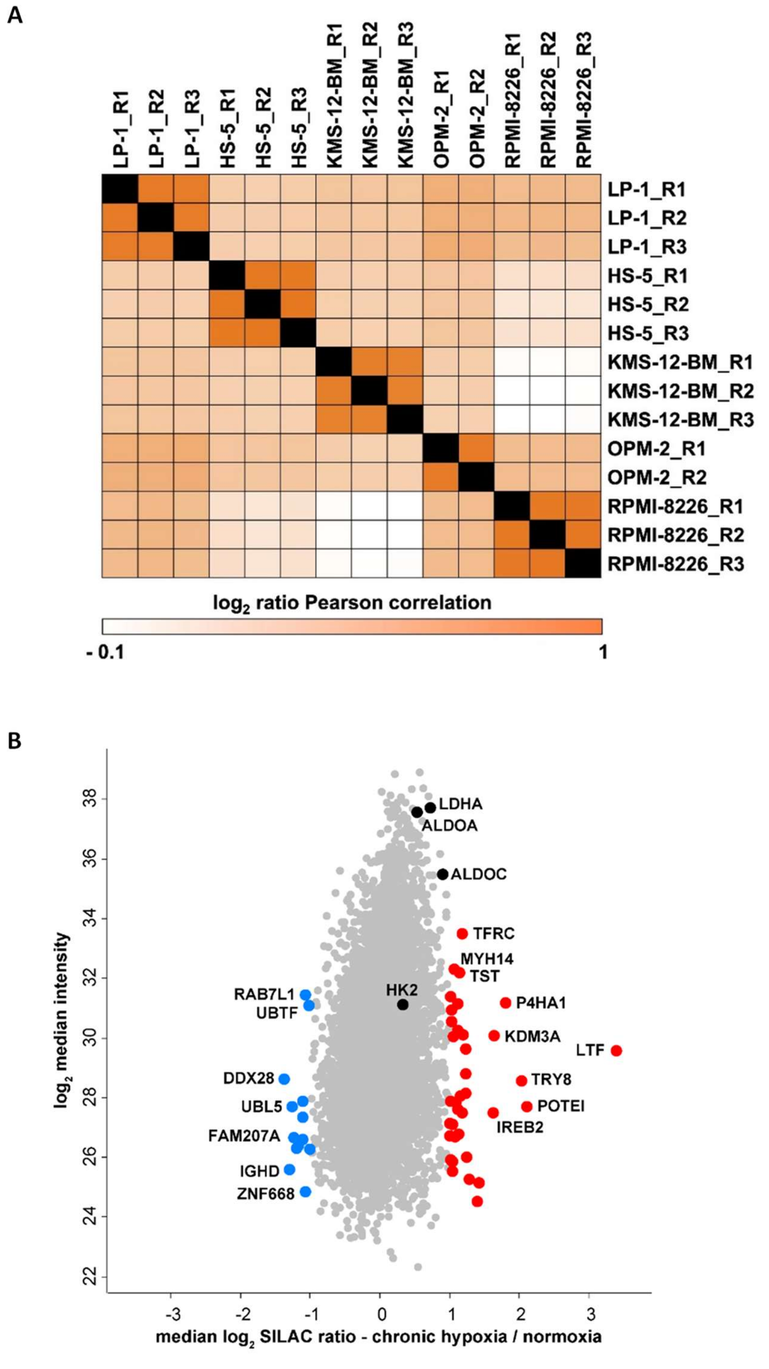

Supplementary Figure S1: Correlation of global proteome profiles and significantly regulated proteins in KMS-12-BM cells: (**A**) The matrix was created by plotting the Pearson correlation coefficient based on the SILAC ratios of each quantified protein in individual LC/MS analyses against all other runs. (**B**) Volcano plot showing proteins significantly regulated under hypoxic conditions KMS-12-BM cells. For statistical analysis, one-sample t-test of the log2-transformed SILAC ratios against zero (no change) was conducted and the *p*-values were adjusted for multiple hypotheses testing (Benjamini-Hochberg FDR < 5%)

Supplementary Figure S2

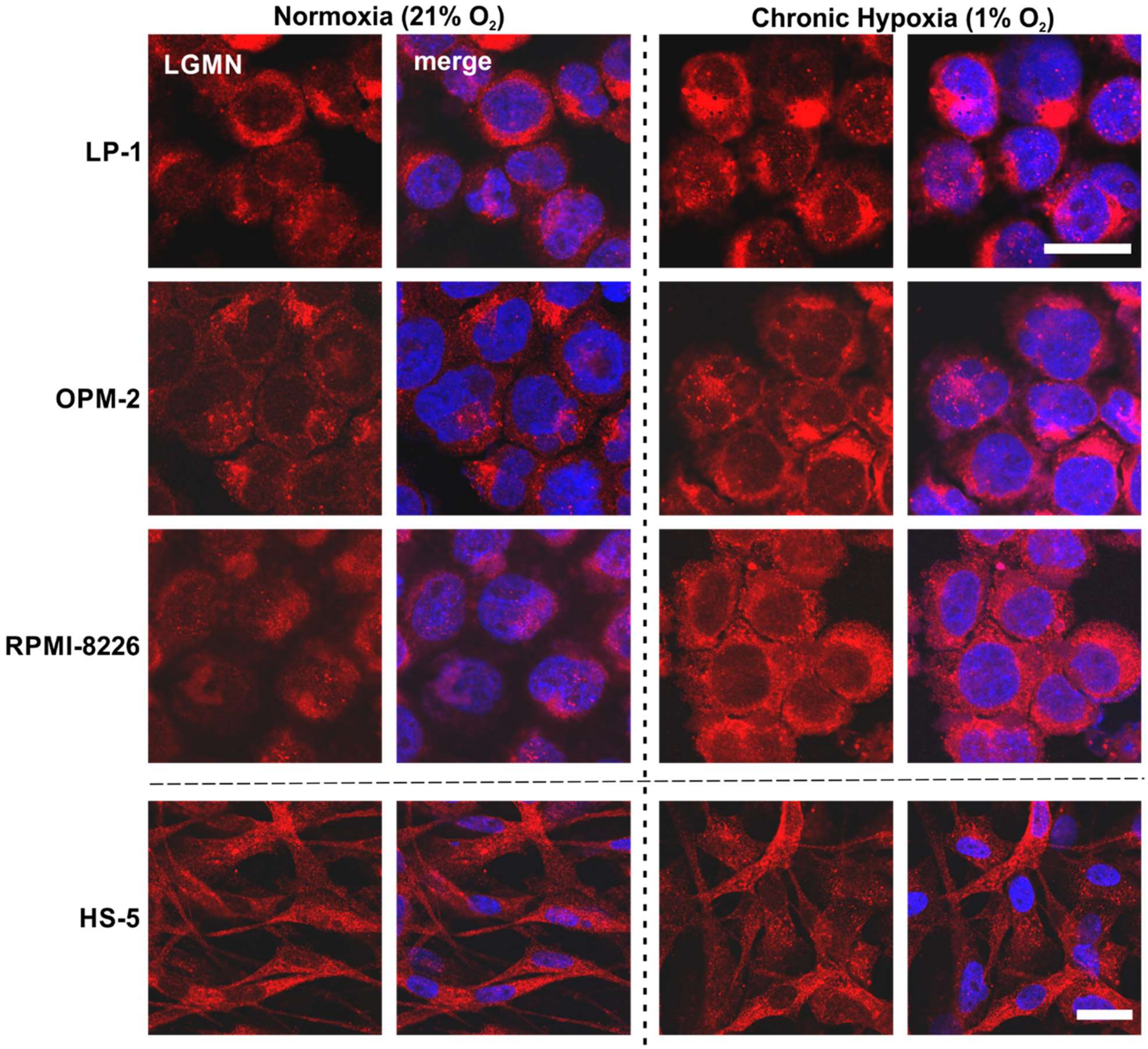

Supplementary Figure S2: Confocal images of the Multiple Myeloma-specific upregulation of LGMN in chronic hypoxia: Three MM cell lines and the stromal cell line HS-5 were cultured under hypoxic conditions (1% O_2) for 7 days and stained for endogenous LGMN. Nuclei were visualized using DAPI. Scale bar = 20 μM

Supplementary Figure S3

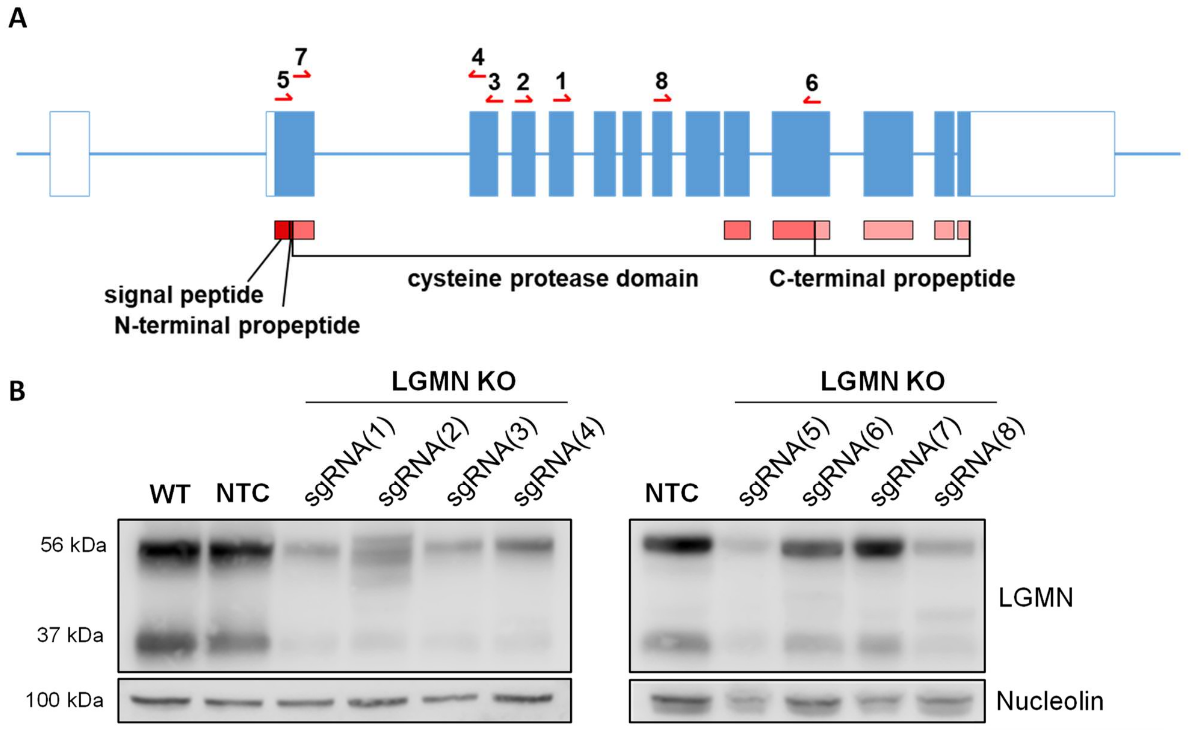

Supplementary Figure S3: Knockout of LGMN in RPMI-8226 cells using eight distinct sgRNAs: (**A**) Schematic view of the gene structure of LGMN. The domains of LGMN protein, and the complementary regions of the sgRNAs used, are indicated. SP, signal peptide; NPP, N-terminal propeptide, AP, activation peptide, LSAM, LGMN stabilization and activity modulation domain (C-terminal propeptide). (**B**) Knockout of LGMN in RPMI-8226 cells using eight distinct sgRNAs. Representative Westernblot of LGMN. Loading control: Nucleolin

Supplementary Figure S4

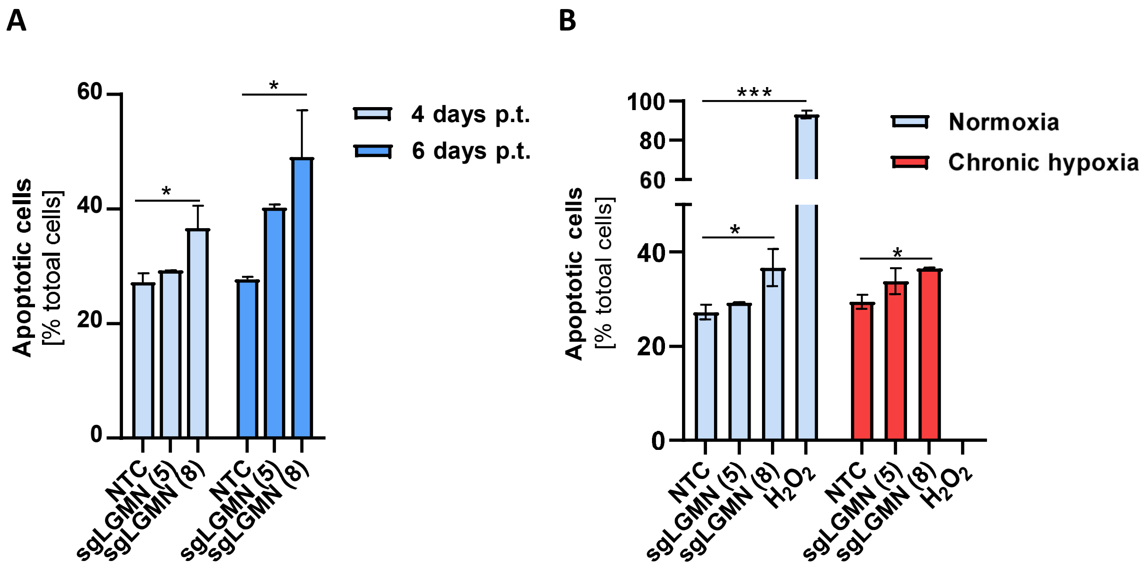

Supplementary Figure S4: Knockout of LGMN in LP-1 cells leads to an oxygen-independent in-duction of apoptosis: (**A**) Annexin V-PE-positive (apoptotic) LP-1 cells [% of total cells] four and six days post transduction with sgRNA NTC, sgRNA LGMN(5) and sgRNA LGMN(8) in normoxic conditions. Bar graphs represent the mean ± SD of two independent experiments. Two-way-ANOVA with Bonferroni's post hoc test. *, $p < 0.05$. Abbreviations: p.t. = post transduc-tion. (**B**) Annexin V-PE-positive (apoptotic) LP-1 cells [% of total cells] four days post transduc-tion with sgRNA NTC, sgRNA LGMN(5) and sgRNA LGMN(8) in normoxic and chronic hypoxic conditions. Bar graphs represent the mean ± SD of two independent experiments. Two-way-ANOVA with Bonferroni's post-hoc test. * $p < 0.05$, *** $p < 0.001$. Data under normoxia are the same as shown in (**A**) for four days post transduction.
